# Decoding Pancreatic Cancer Pain: From Tumor-Nerve Crosstalk to Targeted Analgesic Strategies

**DOI:** 10.7150/ijms.134090

**Published:** 2026-07-29

**Authors:** Daqiang Zhao, Qingwen Hu, Hanxuan Wang, Yu Huang, Yuqian Li, Yu Qian, Li Gao, Qian Ding, Yi Zhun Zhu

**Affiliations:** 1 School of Pharmacy, Faculty of Medicine, Macau University of Science and Technology, Avenida Wai Long, Macau SAR, China.; 2 Laboratory of Drug Discovery from Natural Resources and Industrialization, Macau University of Science and Technology, Avenida Wai Long, Macau SAR, China.; 3 Department of Anesthesiology, Pain Medicine, Palliative Care, Sichuan Taikang Hospital, No.881 Xianghe 1st Street, Huayang Community, Tianfu New Area, Chengdu, Sichuan, P.R. China.; 4 Department of Cardiology, Zhuhai People's Hospital and 1st Affiliated Hospital of Macau University of Science and Technology, Zhuhai, China.

**Keywords:** pancreatic cancer pain, perineural invasion, tumor-nerve interactions, mechanism-based pain therapy

## Abstract

Pancreatic cancer (PC) remains one of the most lethal malignancies, with pain being a prevalent and debilitating symptom that severely impairs patients' quality of life and independently predicts poorer survival. While historically attributed to tumor mass effects, the mechanisms driving PC pain are far more complex. This review aims to synthesize the current understanding of the intricate biological underpinnings of PC-associated pain and discuss the paradigm shift towards mechanism-based therapeutic strategies. The pathogenesis of PC pain is fundamentally driven by dynamic tumor-nerve interactions. A central mechanism is perineural invasion (PNI), a pathologic hallmark wherein cancer cells infiltrate the perineural space, establishing a specialized "neural niche." Within this niche, bidirectional crosstalk involving cancer cells, Schwann cells, immune cells, and stromal components, which is mediated by neurotrophins, chemokines, and neurotransmitters, drives neural remodeling, neuroinflammation, and peripheral nociceptor sensitization. These peripheral signals subsequently induce central sensitization within the spinal cord and brain, perpetuating chronic, refractory pain. While conventional analgesics and interventions like celiac plexus neurolysis remain the mainstay, their relief is often incomplete and transient, as they fail to address these underlying biological drivers. A therapeutic paradigm shift is underway, moving from symptomatic palliation towards mechanism-based interventions. Emerging strategies, including molecularly targeted inhibitors, neuromodulation, and precision nanomedicine platforms, aim to directly disrupt the fundamental neurobiological pathways of pain. By targeting tumor-nerve crosstalk, neuroinflammation, and pathological neural plasticity, these approaches hold the potential to achieve more effective and durable analgesia, ultimately improving the lives of patients suffering from pancreatic cancer.

## 1. Introduction

Pancreatic cancer (PC) is one of the most lethal malignancies worldwide with a rapidly increasing global burden 1-3. During the last 25 years, it has more than doubled in incidence, due to the ageing of the population and the increasing prevalence of established risk factors, such as smoking, obesity, type 2 diabetes and excessive alcohol consumption 4. These factors encourage chronic inflammation of the pancreas and cumulative genetic damage, which promotes malignant transformation 5. Consequently, both the incidence and mortality of PC have increased steadily in many areas. In the USA, about 64,050 new cases and 50,550 deaths were estimated in 2023, making PC the third leading cause of cancer-related mortality 1. Outcomes are similarly poor in Europe where median survival is about 4.6 months and 5-year survival rates are less than 10% 6.

**Figure 1:**
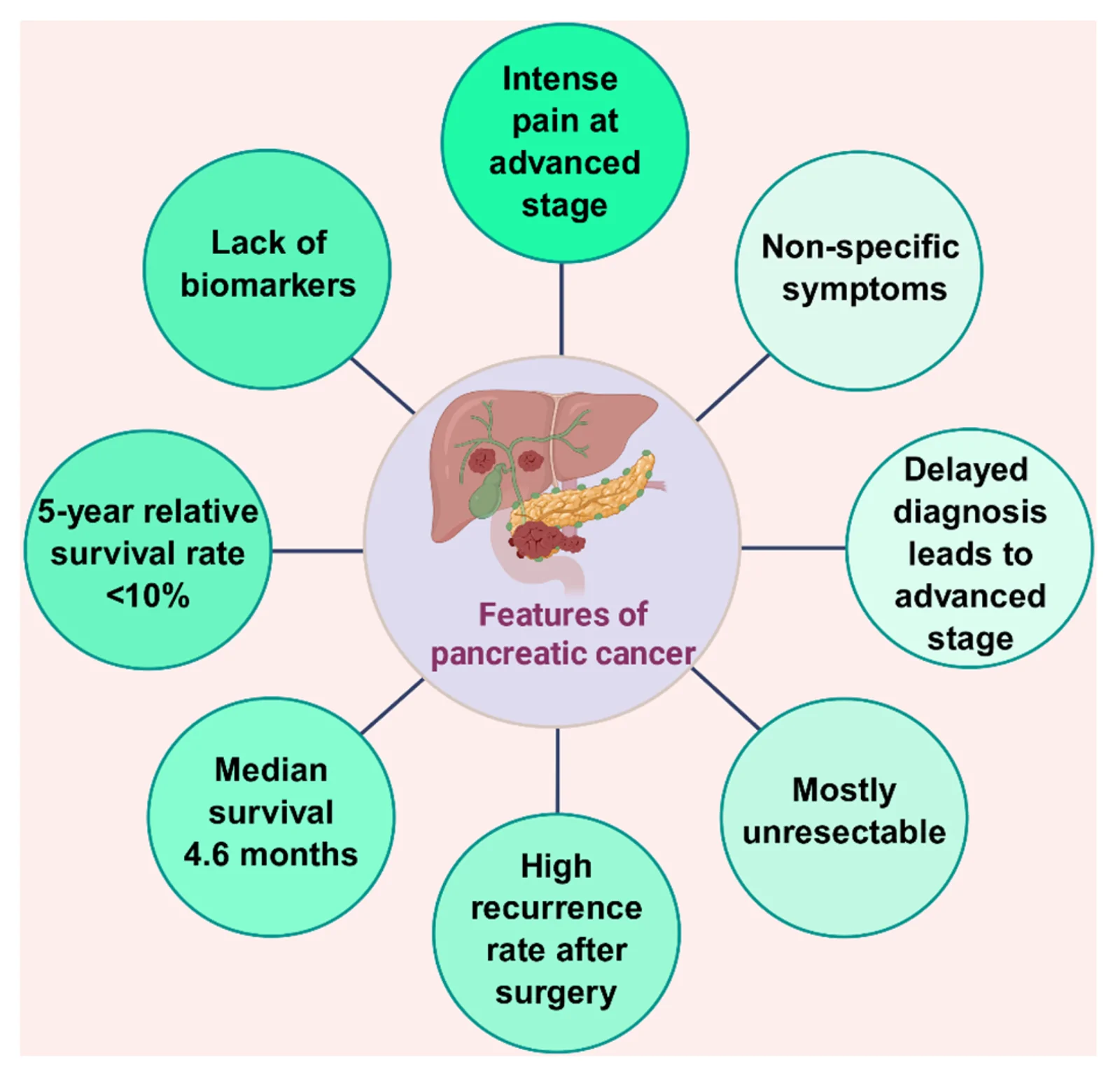
** Features of pancreatic cancer.** Pancreatic cancer commonly presents with non-specific symptoms and lacks reliable early biomarkers, leading to delayed diagnosis and advanced-stage disease in most patients. As a result, many tumors are unresectable at diagnosis, and recurrence after surgery remains frequent. The disease is associated with poor survival, including a median survival of only several months in advanced cases and a 5-year relative survival rate of less than 10%. Severe pain is common in advanced-stage pancreatic cancer and represents an important factor affecting quality of life.

The dismal prognosis of PC is largely due to delayed diagnosis because early stage disease is usually characterized by vague and non-specific symptoms 7. As a result, over 80% of patients are diagnosed with the disease in advanced stages, and only 15-20% are suitable candidates for potentially curative surgical resection; overall median survival is less than one year 7. Unintentional weight loss and cachexia are common at the time of diagnosis 8, 9. Tumors of the head of the pancreas often cause painless obstructive jaundice, while tumors of the body and tail often present later, often with abdominal or back pain and severe cachexia 8, 10, 11. Additional non-specific symptoms include anorexia, fatigue, dyspepsia and in some cases new onset diabetes mellitus 8, 11-14. Pain is a major and clinically important aspect of PC and significantly impairs quality of life and tolerance to anticancer therapies 15-17. At the time of diagnosis, pain is reported in 50-66% of patients and rises to more than 80% in advanced disease 18-20. Pain severity is related to the tumor progression 15, 21, and the greater the intensity of pain, the worse the prognosis, the higher the opioid consumption, and the lower the overall survival 22, 23 (Figure 1).

Pain is a hallmark of PC and results from activation of a dense network of sensory neurons innervating the tumor; its pathogenesis is complex and multifactorial 24. Nociceptive signaling is triggered by noxious stimuli and is conducted by peripheral sensory neurons (unmyelinated C fibers and thinly myelinated Aδ). These nociceptors release glutamate as the main excitatory neurotransmitter, and neuropeptides such as substance P (SP) and calcitonin gene-related peptide (CGRP) mediate spinal transmission further and in the tumor microenvironment. There are both visceral and somatic components of PC-related pain 25. Visceral pain occurs due to pancreatic ductal obstruction, intraductal hypertension, invasion of surrounding organs or malignant ascites, while somatic pain is caused by tumor extension to the peritoneum, retroperitoneum or bone 17, 21, 26-28. Nociceptive signals are transmitted to the central nervous system mostly through the sympathetic nervous system, the splanchnic nerves and celiac plexus 29. Perineural invasion (PNI), a pathologic hallmark of PC characterized by infiltration of malignant cells along peripheral nerves, is associated with both local tumor spread and neuropathic pain 15, 26, 28, 30-32. Neural involvement is present even in early stages of disease, including small T1 tumors, indicating the prominent neurotropism in PC cells 33. Consistent with this early neurotropic behavior, Schwann cells and other nerve-associated glial cells have been seen around preinvasive pancreatic intraepithelial neoplasia suggesting that tumor-nerve interactions may occur prior to overt malignant transformation 34.

Bidirectional crosstalk between pancreatic tumors and the nervous system promotes reactive neural hyperplasia, chronic inflammation, stromal remodeling and increased neuronal excitability in the tumor microenvironment 23. Clinically, these processes are expressed as deep, prolonged abdominal pain that is frequently resistant to normal analgesic treatments 17, 35, 36. Accordingly, management of pain associated with PC is especially difficult due to its multifactoriality and neuropathic nature. Although opioid analgesics are still first-line therapy, even high-dose regimens often do not provide complete or sustained relief, while dose-limiting adverse effects further increase the burden of disease on the patient 37-46. Similarly, interventional procedures such as celiac plexus neurolysis and splanchnic nerve blockade only provide short-term palliation 47. Consequently, many patients suffer from severe and persistent pain despite maximal standard of care treatment, making the need for mechanism-based therapeutic strategies targeting the biological drivers of PC-associated pain particularly urgent.

Rather than regarding pancreatic cancer (PC) pain as a nonspecific consequence of tumor bulk, this review adopts a pain-centered framework in which pain evolves through several interconnected stages. In the initiation phase, pancreatic duct obstruction, local inflammation, tissue distortion, and early neural remodeling activate visceral afferents and generate deep abdominal pain. As the disease progresses, tumor‒nerve crosstalk, perineural invasion (PNI), and neuroimmune interactions drive peripheral sensitization, neurite sprouting, and neuropathic transformation through mediators such as NGF, GDNF-family ligands, chemokines, TRPV1, and neuropeptides 23, 26, 30, 32, 48-60. Persistent nociceptive input then promotes central sensitization within the spinal cord and brain, contributing to pain chronification, hyperalgesia, allodynia, and incomplete opioid responsiveness. Within this framework, molecular and cellular pathways are discussed according to their roles in shaping clinically relevant pain phenotypes, including visceral pain, neuropathic pain, referred back pain, and chronic refractory pain.

## 2. Clinical pain phenotypes and a pain-centered conceptual framework for pancreatic cancer pain

Pancreatic cancer pain is clinically heterogeneous rather than a single uniform symptom. For clinical understanding, it can be divided into four partially overlapping phenotypes: (i) visceral nociceptive pain, which is mainly related to pancreatic duct obstruction, capsular distension, ischemia, inflammation, and invasion of adjacent visceral organs^15^, ^17^, ^21^, ^25^-^27^, ^29^, ^61^, ^62^; (ii) neuropathic pain, which is mainly caused by perineural invasion, perineural neuritis, nerve injury, and peripheral sensitization 16, 23, 26, 63-68; (iii) referred or somatic back pain, which is associated with retroperitoneal extension, peritoneal irritation, or bone involvement 17, 21, 25-27; and (iv) centralized or chronic refractory pain, which is sustained by central sensitization, impaired pain modulation, and possible opioid-related maladaptive plasticity 36, 48, 66, 69-76. These pain phenotypes are not completely separated from each other, and they often coexist or change during disease progression 15, 16, 21, 48, 50, 63. In early stage, tissue distortion and inflammatory signaling activate pancreatic afferents and generate deep, poorly localized visceral pain 15, 50, 61. With progression, neurotrophins, chemokines, ion-channel remodeling, and neuropeptide release lower nociceptor thresholds and promote neuropathic transformation 48, 49, 52, 56, 77-79. In the advanced stage, persistent afferent input, continuous neuroimmune activation, and increased exposure to analgesics may further promote central sensitization, leading to hyperalgesia, allodynia, recurrent breakthrough pain, and reduced responsiveness to opioids 48, 69, 75, 76.

The transition from nociceptive pain to neuropathic and chronic pain in pancreatic cancer is fundamentally determined by the unique neural anatomy of the pancreas. Therefore, before discussing the molecular and cellular mechanisms that drive pain progression, it is necessary to first outline the normal anatomy and physiological functions of the pancreatic nervous system.

## 3. The neural anatomy of the pancreas relevant to pain transmission

Pain signal transmission in the pancreas depends on coordinated sympathetic, parasympathetic, and sensory pathways that connect the pancreas with the peripheral and central nervous systems. These neural circuits provide the anatomical basis for pancreatic nociception, neural remodeling, and pain signaling 80-86.

### 3.1 Extrinsic autonomic nervous system

#### 3.1.1 Sympathetic nervous system (SNS)

Sympathetic innervation originates from preganglionic neurons in the intermediolateral column of the thoracic spinal cord (T5-T9). These fibers travel through the splanchnic nerves to the celiac and superior mesenteric ganglia before projecting to the pancreas 80. Because pancreatic sympathetic fibers converge within the celiac plexus, this structure serves as a major relay for visceral pain transmission and represents an important therapeutic target for celiac plexus block and neurolysis 25, 44, 61, 87-91.

#### 3.1.2 Parasympathetic (Vagus) nervous system (PNS)

Parasympathetic innervation arises from the dorsal motor nucleus of the vagus and reaches the pancreas through the vagus nerve, primarily terminating in intrapancreatic ganglia 80. Vagal afferents project to the nodose ganglion and nucleus tractus solitarius, forming vagovagal circuits that convey visceral sensory information between the pancreas and the brainstem 80.

### 3.2 Sensory afferent nervous system

Pancreatic sensory innervation is mediated predominantly by Aδ and C fibers arising from pseudounipolar neurons in the dorsal root ganglia (DRG) (T6-L2) and nodose ganglia 87. These afferents detect mechanical and chemical stimuli and transmit nociceptive signals to the spinal cord and brainstem. A subset expresses TRPV1 and releases neuropeptides such as substance P and CGRP, which contribute to nociception, neurogenic inflammation, and peripheral sensitization 61, 62, 92, 93. Because most pancreatic cancer pain signals are ultimately transmitted through DRG-associated sensory pathways, these neurons represent a key anatomical substrate for pain generation and chronification.

### 3.3 Intrinsic ENS and intrapancreatic ganglia

**Figure 2:**
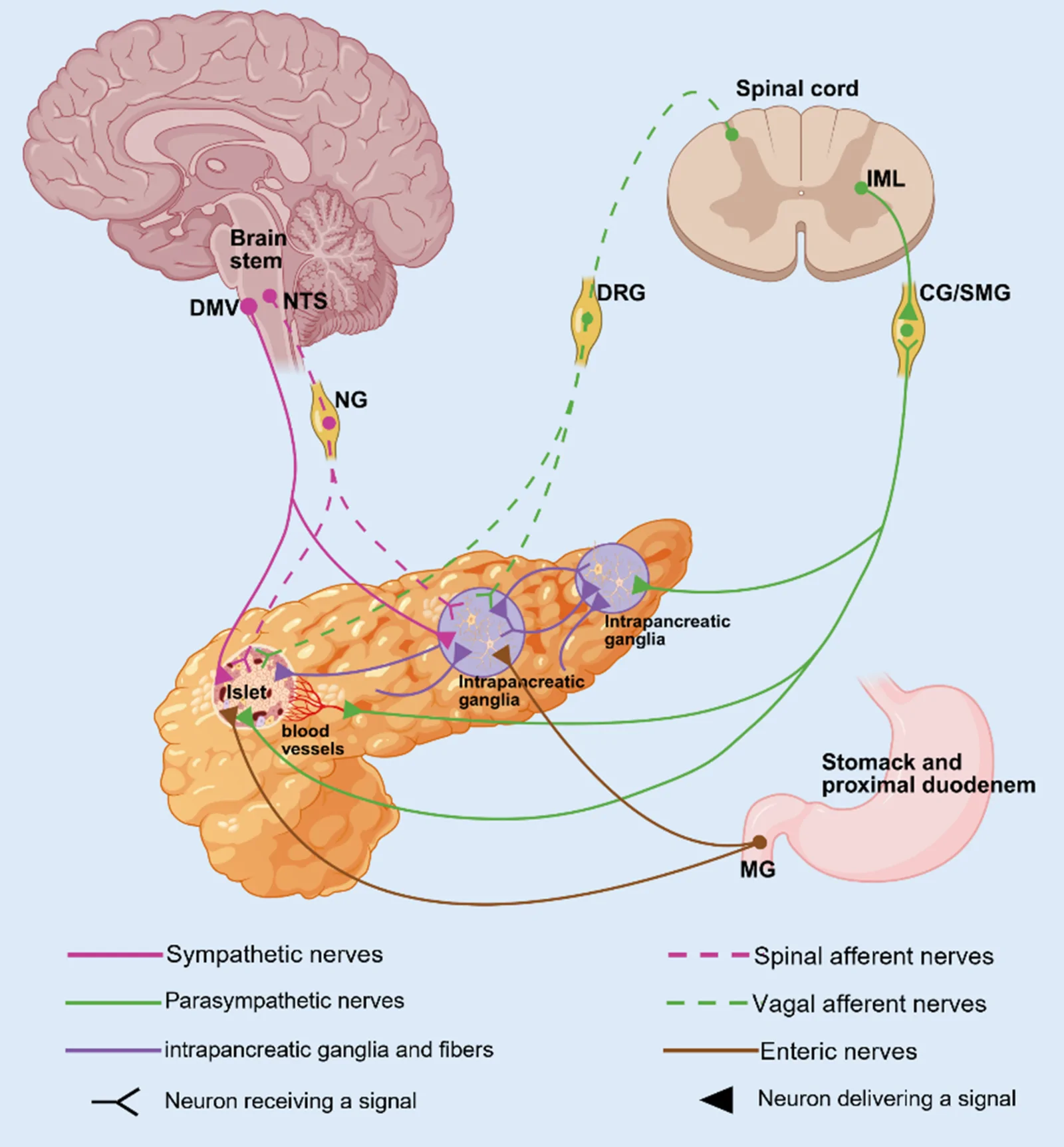
** Neuronal innervation of the pancreas.** The IML of the spinal cord gives rise to sympathetic preganglionic neurons that project via the splanchnic nerves to prevertebral ganglia, chiefly CG and SMG ganglia. From these ganglia, postganglionic fibers innervate the pancreas, preferentially targeting the vasculature and islets, and to a lesser extent the exocrine compartment. Parasympathetic control originates from preganglionic neurons in the DMV, whose axons course in the vagus nerve and synapse mainly within intrapancreatic ganglia; neurons in these ganglia then project to both islet and acinar regions. The NTS forms a vagovagal reflex with the pancreas by integrating input from vagal afferents in the nodose ganglion and shaping DMV efferent output through the vagus nerve, thereby coordinating pancreatic secretory functions. Afferent sensory input reaches the CNS through both vagal and spinal pathways, arising from the nodose ganglion and DRG, respectively. Integration of sympathetic, parasympathetic, and sensory signals occurs within the intrapancreatic ganglia, which also receive modulatory input from the ENS to provide layered regulation. These neural pathways provide the anatomical basis for pancreatic nociception, tumor-nerve crosstalk, neural remodeling, and pain transmission in pancreatic cancer. Abbreviations: IML, intermediolateral cell column; CG, celiac ganglia; SMG, superior mesenteric ganglia; DMV; NTS; ENS.

Intrapancreatic ganglia integrate sympathetic, parasympathetic, sensory, and enteric inputs and communicate with duodenal enteric circuits 48, 86, 94, 95. Although primarily involved in local neural regulation, they may also participate in sensory processing and neuron-immune interactions within the pancreas (Figure 2).

## 4. Neural alterations in PC

The major neurological effects of PC are nerve fiber hypertrophy and hyperplasia in the pancreas. These changes take place by means of three major mechanisms:

1 axonogenesis, sprouting of new axons;

2 neurogenesis, or the formation of new neurons; and

3 neural remodeling, such as the change in the ratio of autonomic and sensory fibers, and activation of peripheral glial cells 48.

### 4.1 Axonogenesis and tumor-nerve crosstalk

Axonogenesis, the pre-neoplastic process of intrapancreatic axon formation, allows for two-way communication between tumor cells and nerves. This process is stimulated by tumor-derived neurotrophins, nerve growth factor (NGF), brain-derived neurotrophic factor (BDNF), and NT-3/NT-4/5, that bind Trk and p75^NTR^ receptors located on the axonal terminals, which promote directed ingrowth of sympathetic, sensory, and cholinergic fibers into the tumor 50, 96-98. These neurotrophic factors promote axonal sprouting and nerve infiltration, which provides a microenvironment that is conducive both to tumor progression and pain signaling 48.

### 4.2 Neurogenesis and nerve outgrowth in the tumor microenvironment

Neurogenesis, the creation of new neurons from progenitor or cancer stem cells, is different from axonogenesis, the growth of axons from pre-existing neurons. Pancreatic tumor cells induce nerve growth and penetration through neurogenesis and axonogenesis 99.

### 4.3 Neural remodeling and altered autonomic balance

Neural remodeling in PC entails two major alterations: 1 a shift in the balance of intrapancreatic autonomic and sensory fibers, and 2 changes in the activation state of neighboring glial cells 48. Sympathetic innervation is significantly diminished as a result of neural invasion by the tumor, whereas the cholinergic fibers are relatively spared 100. This denervation is accompanied by an increase in nociceptive fibers, as is the case in chronic pancreatitis, where an increased SP and CGRP are involved in the heightened pain signal 101.

Schwann cells are important contributors to neural remodeling, pain persistence and tumor development. Normally supporting axonal myelination and neural maintenance, they are activated in pancreatic tumors, and form dynamic tracks that promote cancer cell migration and invasion 65. Non-myelinating Schwann cells also produce growth factors (midkine (MDK) and interleukin-1α (IL-1α) that promote tumor growth and inflammatory remodelling of the stroma, whereas the lncRNA PVT1 contributes to immune evasion 102, 103. These adaptive Schwann cell responses create a self-reinforcing tumor-nerve network that is responsible for both neural remodeling and disease progression 102, 103.

## 5. PNI in PC

**Figure 3:**
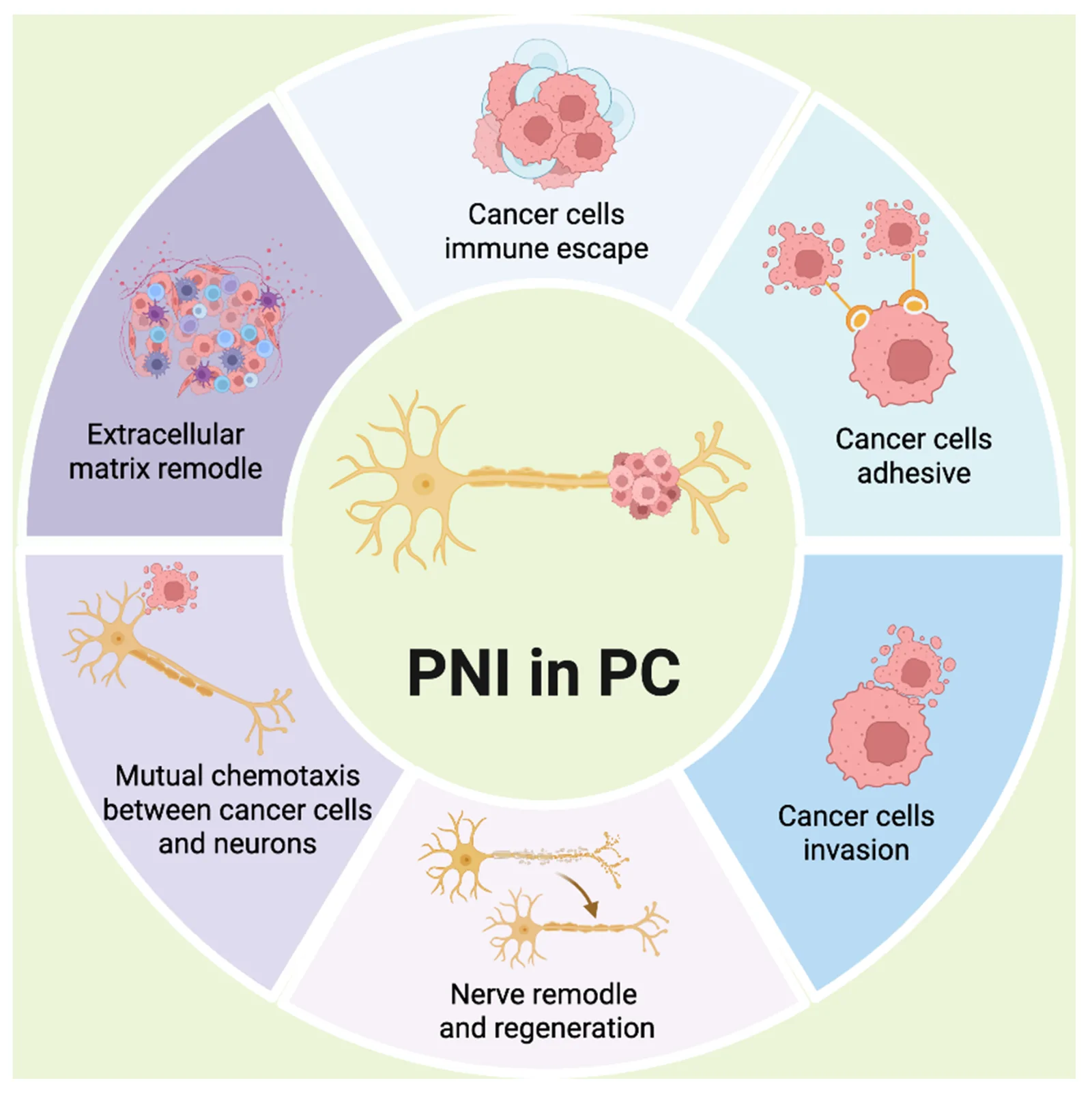
** The process of PNI in pancreatic cancer.** Perineural invasion (PNI) in pancreatic cancer (PC) is a dynamic and multi-step process involving reciprocal interactions between cancer cells, nerves, stromal cells, immune cells, and the extracellular matrix. During this process, pancreatic cancer cells acquire increased adhesive and invasive capacities, migrate toward nerves through mutual chemotactic signaling, and infiltrate the perineural space. At the same time, nerve injury, nerve remodeling, and regenerative responses provide a permissive neural niche for further tumor spread. Remodeling of the extracellular matrix and immune escape further support cancer cell migration and survival around nerves. These coordinated events promote local tumor progression, neural injury, peripheral sensitization, and PNI-associated pain in PC.

**Figure 4:**
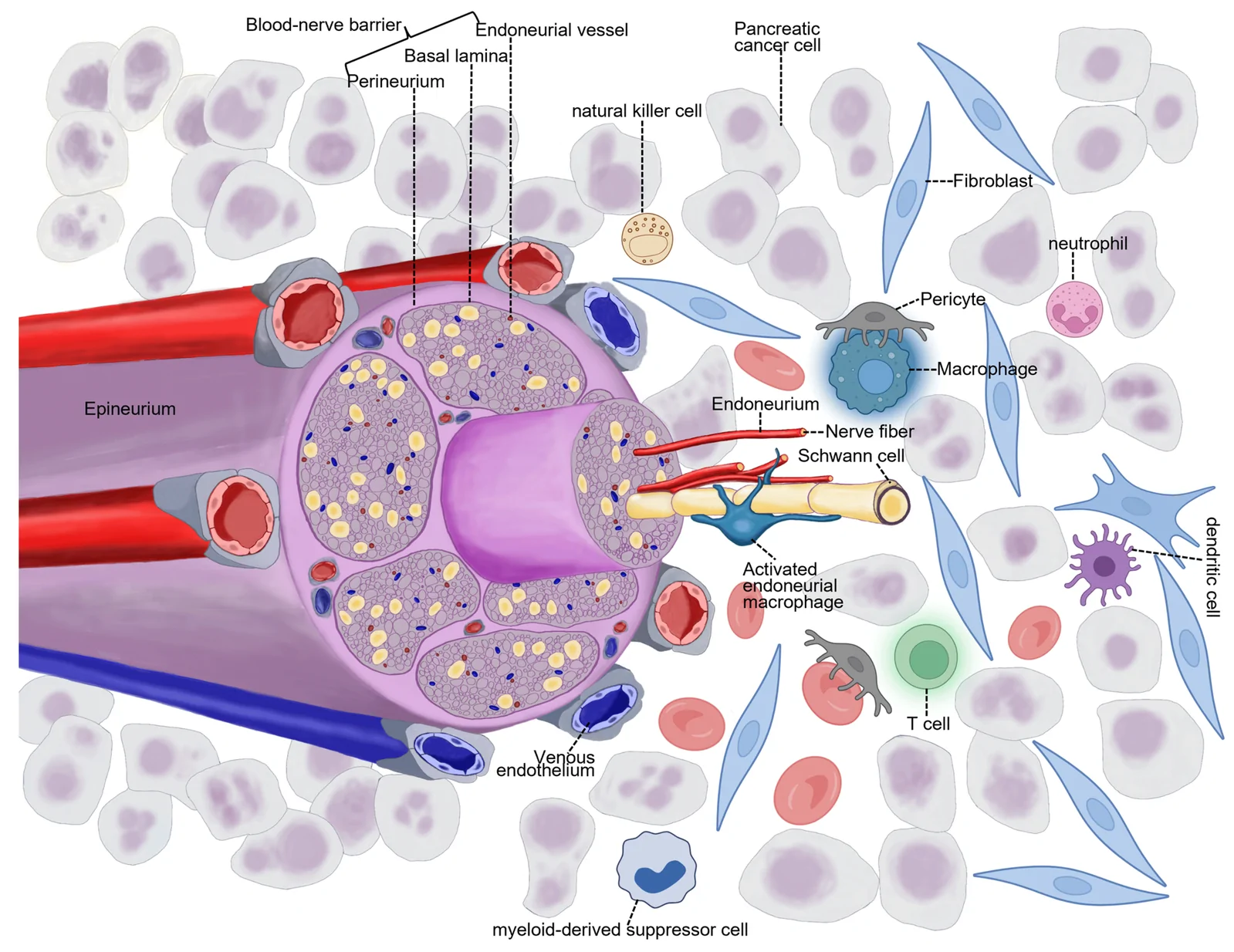
** Structure of the neural niche.** In pancreatic cancer, the neural niche is a specialized compartment within the perineural invasion microenvironment that integrates neural, immune, stromal, vascular, and cancer cell components. Neural elements mainly include neurons, nerve fibers, and Schwann cells, whereas immune components include dendritic cells, T cells, natural killer cells, macrophages, neutrophils, and myeloid-derived suppressor cells. Many of these cells express receptors for neurotransmitters or neuropeptides and can respond to neuronal signals. Anatomically, peripheral nerves are composed of axons and Schwann cells organized into fascicles within the endoneurium. These fascicles are surrounded by the perineurium, and the entire nerve trunk is enclosed by the epineurium. Nerve fibers may be myelinated or unmyelinated, and Schwann cells, together with resident macrophages, participate in debris clearance, nerve repair, and regeneration. In pancreatic cancer, repair-like Schwann cells can be hijacked by cancer cells and used as migratory tracks, thereby transforming perineural invasion from a simple route of local spread into a dynamic structural and functional interface between nerves and tumor cells. The blood-nerve barrier is formed mainly by endoneurial microvessels and the surrounding perineurium, both of which contain abundant tight junctions. This barrier is further supported by pericytes along capillary walls, which are important for vessel formation and maintenance of barrier integrity 256. These anatomical structures create a protected space for axons, Schwann cells, and other peripheral nerve cells, while restricting the entry of circulating molecules and immune cells 257, 258. When the blood-nerve barrier is disrupted, tumor cells and inflammatory cells can enter the endoneurial space, where they damage neural and glial elements, promote neuroinflammation, and remodel the local extracellular matrix 259. Through dense bidirectional signaling among cancer cells, nerves, Schwann cells, immune cells, and stromal cells, these structural changes establish a dysregulated neural niche that promotes further perineural invasion, sensitization of nociceptive fibers, and persistent neuropathic pain in pancreatic cancer.

PNI, the infiltration of tumor cells into the perineural space, is a feature of PC 64. PNI is present in up to 86% of resected specimens and is linked to aggressive histology, early recurrence and decreased disease-specific survival, especially in the presence of large-caliber nerves (≥3 mm) or nerve necrosis 104. Far from being a passive pattern of spread, PNI is a biologically active process that is driven by dynamic tumor-nerve interactions (Figure 3). It acts as a conduit for local dissemination and a specialized neural niche of nerves, Schwann cells, cancer cells, stromal constituents, and immune cells, protected by a blood-nerve barrier 25, 105. Within this niche, bidirectional signaling is mediated by trophic and chemotactic cues 30: tumor cells secrete neurotrophins, chemokines and axon-guidance molecules that promote neuritogenesis, nerve hyperplasia and remodeling, while Schwann cells and sensory neurons secrete GDNF family ligands to facilitate tumor adhesion, directed migration and invasion along nerves 104. Nerve injury and demyelination also trigger repair Schwann cells and attract macrophages, which remodel the extracellular matrix (ECM) and strengthen axon-cancer alignment 25, 105 (Figure 4). The resultant inflammatory and immunosuppressive microenvironment contributes to local tumor progression, neural injury and sensitization and to neuropathic pain initiation and maintenance.

Because PNI is a major driver of neural injury, sensitization, and pain generation in pancreatic cancer, the molecular pathways discussed below are considered in terms of their contributions to pain biology as well as neural invasion. Many of these mediators influence nociception, peripheral sensitization, neuroinflammation, neuropathic transformation, and pain chronification.

### 5.1 Principal molecular regulators in PNI

#### 5.1.1 Neurotrophic factors and corresponding receptors

Chemotactic gradients of neurotrophic factors produced by neurons, stromal cells, and pancreatic tumor cells are important drivers of tumor-nerve interaction in PC. The NGF-TrkA axis, which is well known in peripheral nerve injury, is upregulated in PC and activates downstream RAS/ERK and PI3K/AKT signaling pathways 106, 107. These pathways promote tumor cell proliferation, migration, invasion, and extracellular matrix degradation through mediators such as MMP-2, thereby facilitating neural infiltration and PNI 106-110. Sonic hedgehog (Shh) signaling can further increase NGF expression in pancreatic stellate cells and neurons, linking stromal activation with neural remodeling and tumor-nerve crosstalk 55. Beyond its established role in tumor growth and neural invasion, the NGF‒TrkA axis should be regarded as an important pain-generating pathway in PC 48, 49, 55, 56.

BDNF acting via TrkB and low-affinity coreceptor p75^NTR^ is also upregulated in PC leading to tumor proliferation, invasiveness, and possible PNI 26, 111, 112. BDNF/TrkB signaling has reinforcing tumor-nerve attraction effects 113-115 , while p75^NTR^ has context-dependent inhibitory or facilitatory effects on PNI 110, 116.

The glial cell line-derived neurotrophic factor (GDNF) and its co-receptors GFRα1 and RET represent a significant pathway 117. GDNF signaling enhances tumor invasiveness by promoting ECM adhesion, inducing filopodia formation, and elevating MMP-9 expression 49, 118-120. Notably, pancreatic cancer-derived exosomal lncRNA XIST is transferred to neural cells, where it sponges miR-211-5p to de-repress and upregulate GDNF; elevated GDNF activates RET signaling in pancreatic cancer cells and drives perineural invasion, whereas XIST knockdown reduces neural GDNF, diminishes RET phosphorylation, and attenuates PNI in vitro and in vivo 121. Similarly, artemin (ARTN), a member of the GDNF family, operates through GFRα3/RET signaling to induce neural hypertrophy and cancer cell migration within the tumor stroma 122, 123.

Neurotrophin-related pathways provide important molecular cues for neural invasion, tumor-nerve communication, and the formation of a pain-permissive perineural niche.

#### 5.1.2 The MDK family

The MDK family, which includes MDK and pleiotrophin (PTN), is involved in PNI in PC. Both are overexpressed and signal through a neuronal receptor, syndecan 3 (SDC3) 124. Elevated MDK is associated with PNI and worse outcomes 124, whereas PTN from tumor cells induces nerve proliferation via SDC3 125, 126. Upregulated SDC3 may also contribute to the aggregation of cancer cells around nerves, worsening neural injury and PNI 125, 126.

#### 5.1.3 Chemokine signaling

Chemokine gradients play a critical role in the direction of PC cell migration towards nerves. The CXCL12/CXCR4 axis stimulates chemotaxis, upregulates MMPs and NGF, and neural invasion 53, 127. Similarly, CX3CL1/CX3CR1 promotes adhesion and directional migration of CX3CR1-positive tumor cells to fractalkine-expressing neurons and endothelial cells 54, 128. Other chemokine circuits also define the tumor-nerve microenvironment. CCL2/CCR2 signaling from nerves, Schwann cells and stromal cells recruits monocytes/macrophages and enhances tumor cell motility, inhibition of which decreases PNI in preclinical models 129, 130. Neuron-derived chemokines CCL21 and CCL10, CCR3 and CCR7 signaling mediate chemotaxis of cancer cells to sensory nerves and neural remodeling; blocking these pathways reduces abdominal hypersensitivity and nerve hypertrophy without affecting T-cell or neutrophil infiltration. Clinically, high levels of CXCR3 and CCR7 are associated with higher levels of pain 52. Interfering with these axes could disrupt tumor-nerve chemotaxis and adhesion, limiting PNI in PC 131. Regarding pain generation and maintenance, chemokine signaling is important because it links neural tropism to sensory amplification 52, 128. By recruiting immune cells, promoting neurite‒tumor attraction, and reinforcing neurotrophic signaling, chemokine pathways help convert local tumor‒nerve interaction into clinically apparent abdominal hypersensitivity and neuropathic pain 17, 23, 52, 60. Clinically, pathways related to sensory neuron recruitment and nerve hypertrophy may be more relevant to patients who present with back-radiating pain, increased local tenderness, and gradually increasing opioid requirements. 8, 22, 23, 69.

#### 5.1.4 Cell adhesion molecules

The MUC1-MAG interaction is more likely to contribute to pain indirectly through its effects on tumor-nerve adhesion and neural invasion 25, 102, 105, 132, 133. By strengthening tumor-Schwann cell adhesion and prolonging tumor-nerve interaction, this pathway may intensify neural injury and sustain the local microenvironment required for neuropathic pain maintenance 65, 102, 105, 132. However, current evidence does not yet show that MUC1-MAG independently defines a specific pain phenotype, and its contribution to pain should therefore be discussed conservatively.

#### 5.1.5 Matrix metalloproteinases (MMPs)

MMP-2 and MMP-9 are responsible for ECM and perineurial remodeling at the tumor-nerve interface in PC, which leads to nerve sheath invasion 134, 135. NGF-TrkA activates MMP-2, GDNF-RET activates MMP-9, and the two factors promote tumor invasiveness 136 and prolonged perineural migration 134, 136-138.

#### 5.1.6 Neurotransmitters

In PC, neurotransmitters play a key role in PNI. Norepinephrine (NE) activates the ADRB2-PKA-STAT3 pathway that causes release of NGF and drives tumor migration to nerves 139, 140. Acetylcholine (ACh) from the vagus nerve induces an immunosuppressive microenvironment by inhibiting CCL5 and decreasing the recruitment of CD8⁺ T-cells through the action of HDAC1, which promotes PNI and tumor growth 141. Peptidergic neurotransmitters also participate in tumor-nerve communication. SP and its receptor NK1R are upregulated in pancreatic cancer and the perineural niche, where they promote cancer cell migration, invasion, epithelial-mesenchymal transition, and directional growth toward nerves 58, 142, 143. Co-released CGRP may further enhance neuronal hyperexcitability and tumor-nerve signaling 48, 59, 93, 144. These findings suggest that neuropeptide signaling contributes to both PNI progression and pain amplification, although its direct clinical value as an analgesic target in pancreatic cancer remains to be validated 16, 48, 58, 59, 143, 144.

#### 5.1.7 Post-transcriptional and non-coding RNA-mediated regulation of PNI

Recent studies on post-transcriptional regulation have expanded our understanding of how pancreatic cancer cells interact with nerves, but their direct relevance to pain should be interpreted carefully. NAT10-mediated ac4C modification of ITGB5 and the RBMS1‒circNFIB‒L1CAM axis appear to act primarily by strengthening tumor-nerve adhesion, facilitating directional migration, and increasing the efficiency of PNI 145, 146. Their relevance to pain is mechanistically plausible but currently supported only by indirect evidence. These pathways may exacerbate pain by prolonging tumor-nerve contact, exacerbating nerve injury, and promoting downstream nociceptor sensitization 23, 26, 48, 55, 56, 65, 105. Nevertheless, direct links between these pathways and specific pain phenotypes (such as neuropathic pain severity, visceral hypersensitivity, or pain chronification) remain poorly characterized 16, 17, 23, 69. In this review, these pathways are therefore regarded as upstream modulators of pain permissiveness, whereas their roles as direct mediators of pancreatic cancer pain remain to be established.

### 5.2 Cellular mediators in the PNI

PNI is dependent on dynamic crosstalk between cancer cells, Schwann cells, macrophages, PSCs, and CAFs. Schwann cells recruited by NGF/p75^NTR^ signal undergo a repair-like phenotype that directs tumor migration 102. They are upregulated for c-Jun, SOX10 and GAP43 147, 148, creating tumor-tracking pathways, which are further stimulated by tumor-derived extracellular vesicles enriched in IL-8 and CCL2 to secrete neurotrophins (e.g. GDNF) and MMP-9, creating a self-sustaining neuroinvasive loop 147. Schwann cells promote adhesion by MUC1-MAG and L1CAM and are regulated by Vitamin D binding protein (GC) (through FAK/MAPK) and transforming growth factor-β (TGF-β), which also induces ECM remodeling and epithelial-to-mesenchymal-like transitions in tumor cells 132, 133, 146, 149-154. Tumor-Associated Macrophages (TAMs) infiltrate invaded nerves, correlate with poor prognosis, and reinforce Schwann cell activation by bFGF/PI3K/AKT→c-Myc/GFAP and IL-33 signaling in a feed-forward loop that amplifies neural invasion 67. Schwann cell chemokines, including CXCL1/2 and galectin-3, attract monocytes and macrophages, thereby maintaining an immunosuppressive and pro-inflammatory perineural niche that facilitates neural invasion, neuroinflammation, and neuropathic pain 67, 147. Schwann cells should be viewed as active participants in pain generation rather than merely invasion-permissive cells. In response to tumor-derived signals and nerve injury, Schwann cells adopt a repair-like phenotype that guides cancer cell migration and also contributes to demyelination, neurotrophic production, neuroinflammation, and maladaptive axonal remodeling 48, 49, 65, 66, 100, 102. These changes increase ectopic neuronal activity and may contribute to the maintenance of neuropathic pain in pancreatic cancer 23, 60, 66. Therefore, Schwann cell-mediated mechanisms may be closely associated with pain persistence and chronification, particularly in patients presenting with radiating abdominal or back pain and neuropathic symptoms.

PSCs secrete neurotrophins and matrix proteins (NGF, TGF-β, laminin) to promote neurite outgrowth and chemotaxis via CXCL12/CXCR4 and interact with Schwann cells via CXCL12/CXCR4 and IL-6/STAT3 to remodel ECM and maintain nociceptive signaling 70, 148, 150, 155-159. CAFs, derived in part from PSCs, give rise to axon-guidance molecules (SLIT2, LIF) and NGF, and promote nerve infiltration, Schwann cell growth, immune exclusion, chemoresistance and cancer-associated pain 160-163. SLIT2-ROBO signaling activates N-/β-catenin signaling pathways that are involved in Schwann cell growth, neural remodeling, and context-dependent tumor proliferation, migration, angiogenesis, and apoptosis 160, 161.

**Figure 5:**
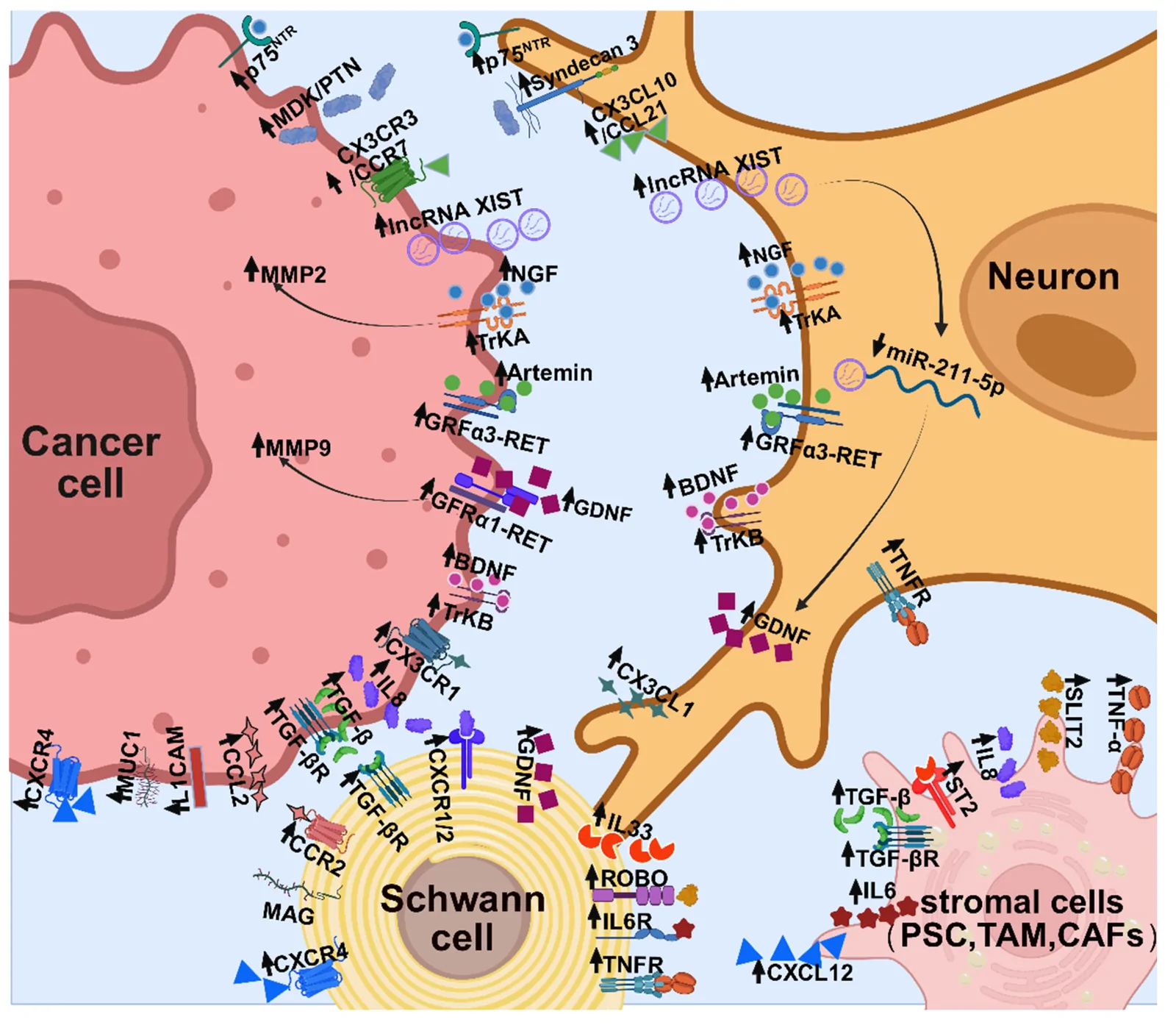
** Major molecules and cellular mediators involved in the process of perineural invasion in pancreatic cancer.** Perineural invasion (PNI) in pancreatic cancer is maintained by dynamic crosstalk among cancer cells, neurons, Schwann cells, stromal cells, and immune cells. Neurotrophic signaling provides important pro-invasive signals. The NGF-TrkA/p75NTR axis, BDNF-TrkB signaling, and GDNF-family pathways, including GDNF-GFRα1-RET and ARTN-GFRα3-RET, promote tumor-nerve interaction and neural invasion. NGF-TrkA mainly induces MMP-2 expression, whereas GDNF-related signaling upregulates MMP-9, thereby promoting extracellular matrix and perineurial degradation and facilitating cancer cell migration along nerves. Exosomal lncRNA XIST may further enhance GDNF signaling by suppressing neuronal miR-211-5p. In the PNI microenvironment, chemotactic and adhesive signals guide and stabilize cancer cell migration along nerves. Neuron-derived CX3CL1 attracts CX3CR1-positive pancreatic cancer cells and Schwann cells toward neurites. CCL2 recruits CCR2-positive cells to the tumor-nerve interface, IL-8 activates CXCR1/2 signaling to enhance directional migration, and stromal CXCL12 forms a chemotactic gradient sensed by CXCR4-positive cells. Adhesion molecules further strengthen tumor-nerve contact: L1CAM promotes cancer cell attachment to nerve fibers, MUC1 binds MAG on Schwann cells, and CX3CL1-CX3CR1 signaling may provide additional adhesion to CX3CL1-positive neurites. Inflammatory and stromal mediators also amplify PNI. TNF-α from tumor-associated macrophages and other myeloid cells activates TNF receptors on Schwann cells and sensory neurons, enhancing NF-κB/MAPK-related expression of chemokines, adhesion molecules, and MMPs. This process promotes perineural inflammation, neural remodeling, and PNI-associated neuropathic pain. In addition, tumor- or stroma-derived TGF-β can induce a repair-like, pro-invasive Schwann cell phenotype, increasing neurotrophic factor and MMP production, promoting extracellular matrix remodeling, and forming cellular tracks that facilitate tumor spread along nerves. Abbreviations: NGF, nerve growth factor; TrkA, tropomyosin receptor kinase A; BDNF, brain-derived neurotrophic factor; TrkB, tropomyosin receptor kinase B; p75^NTR^, p75 neurotrophin receptor; GDNF, glial cell line-derived neurotrophic factor; GFRα1/3, GDNF family receptor-α1/3; RET, rearranged during transfection receptor tyrosine kinase; ARTN, artemin; CX3CL1, fractalkine; CX3CR1, CX3C chemokine receptor 1; CCL2/CCL12, C-C motif chemokine ligand 2/12; CCR2, C-C chemokine receptor 2; CXCR4, C-X-C chemokine receptor 4; TNF-α, tumor necrosis factor-α; TNFR, tumor necrosis factor receptor; IL-6/IL-8/IL-33, interleukin-6/-8/-33; IL6R, interleukin-6 receptor; CXCR1/2, C-X-C chemokine receptors 1/2; MDK, Midkine; PTN, Pleiotrophin; SDC3, Syndecan-3; SLIT2, slit guidance ligand 2; ROBO, roundabout guidance receptor; L1CAM, L1 cell adhesion molecule; MUC1, mucin-1; MAG, myelin-associated glycoprotein; lncRNA XIST, X-inactive specific transcript; miR-211-5p, microRNA-211-5p; MMP-2/9, matrix metalloproteinase-2/-9; ECM, extracellular matrix. TGF-β, Transforming growth factor-β; TGF-βR, Transforming growth factor-β receptor.

Schwann cells, tumor cells (and their EVs), TAMs, PSCs and CAFs communicate bidirectionally through neurotrophins, chemokines and adhesion molecules to promote PNI. Schwann cells: form migratory tracks; produce trophic factors, TAMs: pro-inflammatory signaling, PSCs: chemotactic cues, ECM scaffolds, CAFs: axon-guidance molecules (e.g. SLIT2, LIF). Together, these interactions create a self-reinforcing neurotrophic niche, which encourages neural infiltration, neuropathic pain, and tumor progression in PC 67, 164 (Figure 5).

### 5.3 Pain induced by PNI in PC

Pain from PNI is one of the most debilitating symptoms of PC caused by direct nerve injury, molecular sensitization and maladaptive neuroplasticity at the tumor-nerve interface 63. Reciprocal tumor-nerve and neuro-immune-stromal interactions are responsible for this pain and suggest the need for mechanism-based approaches to better manage pain 26, 48, 49, 165.

The following are the key mechanisms of pain generation in PNI:

#### 5.3.1 Nerve remodeling and damage

Physical disruption of the neural sheath by invading tumor cells is involved in neuropathic pain in PC 64, 166. Tumor-associated hypoxia exacerbates this through HIF-1α induced GM-CSF, which facilitates Schwann cell activation and maladaptive neuroplasticity, enhancing pain signaling 23, 100.

#### 5.3.2 Neurotrophin-driven nociceptive remodeling and ion channel sensitization

Tumor-secreted factors activate sensory neurons by raising neurotrophic and ion channel signaling 24-26, 48, 49, 55, 56, 77, 109. Tumor and stromal cells release Sonic hedgehog (Shh) and chemokines (e.g. CXCL12) that increase nerve growth factor (NGF) and related neurotrophins (GDNF, artemin, BDNF) 32, 48-50, 53, 55, 102, 167. These neurotrophic factors activate TrkA and p75^NTR^ signaling in dorsal root ganglion neurons, which increases TRPV1 expression, enhances neuronal excitability 55-57, 77, 106, 109, 110, 168. The upregulation of TRPV1 decreases the activation threshold of nociceptors and increases Na^+^ and Ca^2+^ influx after noxious stimulation 56, 57, 78, 79, 169, 170. Increased intracellular calcium promotes the release of SP and CGRP from sensory nerve endings. SP binds to neurokinin-1 receptors (NK1R), while CGRP acts on CGRP receptors, including the CLR/RAMP1 complex 58, 59, 62, 93, 142, 144. These receptors can be expressed by neurons, Schwann cells, immune cells, and tumor cells 58, 59, 65, 66, 102, 142, 144. Their activation further enhances neurogenic inflammation and tumor-nerve communication, thereby maintaining nociceptor sensitization and contributing to persistent abdominal and neuropathic pain in pancreatic cancer 17, 26, 48, 58, 59, 62, 93. Chemokines such as CXCL12 work together with neurotrophins to promote nerve sprouting toward the tumor, leading to peripheral sensitization and neuropathic pain features 30, 48, 53, 54, 159, 171.

In experimental models, inhibition of these pathways may reduce pain-related behaviors 55, 56. Anti-NGF antibodies or TrkA inhibitors have been reported to decrease hyperalgesia, while TRPV1 blockers can reduce nociceptor excitability 57, 98, 169, 172-175. NK1R or CGRP receptor antagonists similarly reduce neurogenic inflammation 58, 59, 143. However, clinical trials (e.g. NGF antibodies or TRPV1 antagonists) have had limited success due to side effects or modest efficacy 25, 169, 175, 176. Thus, targeting NGF/TrkA, TRPV1, NK1R, or CGRP signaling may provide a possible therapeutic direction, although its clinical translation remains challenging.

#### 5.3.3 Cellular contributions

Schwann cells interact with pancreatic cancer cells through MUC1-MAG and NGF-dependent signaling, thereby contributing to neuroplastic changes in invaded nerves 34, 66. These interactions may increase the expression of nociceptive mediators in sensory fibers, including TRPV1, substance P, and CGRP 55, 56, 58, 59, 77, 93. In the CNS, NGF and TGF-β activated astrocytes and microglia cause central sensitization by releasing pro-inflammatory cytokines 66, 70. PSCs promote pain through TGF-β mediated NGF, laminin mediated neurite outgrowth, MMP-2/MMP-9 mediated nerve exposure and CXCL12/CXCR4 mediated NGF reinforcement of nerve circuits 53, 110, 159, 177-179.

### 5.4 Pain caused by perineural neuritis in PC

Perineural neuritis in PC is characterized by extensive infiltration of immune cells within hypertrophic nerves and their sheaths and is strongly associated with abdominal pain 23. CD8⁺ cytotoxic T cells, macrophages, and mast cells frequently accumulate around injured or invaded nerves, creating a neuroinflammatory microenvironment that amplifies nociceptive signaling 23, 60, 87, 180. Among these interactions, bidirectional communication between sensory neurons and mast cells plays an important role. Neurotransmitters released from activated nociceptors can induce mast cell degranulation, while mast cell-derived mediators further sensitize adjacent sensory nerve endings 60. This interaction contributes to neuronal dysfunction, nociceptor hyperexcitability, and neuropathic abdominal pain 60. Consistent with this mechanism, mast cell accumulation around invaded nerves has been associated with pain severity, which supports the possible analgesic role of mast cell stabilization or inhibition of degranulation 60, 181. Importantly, perineural neuritis may represent a critical transitional process in which structural nerve involvement gradually develops into active neuroimmune-mediated pain amplification 23, 60, 66. Once immune cells accumulate around hypertrophic or invaded nerves, pain is no longer driven only by mechanical injury, but is further sustained by reciprocal signaling among neurons, mast cells, macrophages, and inflammatory mediators 23, 48, 60, 66, 67. This neuroimmune feed-forward loop may explain why pancreatic cancer pain often becomes disproportionate to tumor burden and progresses to persistent spontaneous pain, hypersensitivity, and reduced responsiveness to conventional analgesics.

## 6. Central sensitization and pain chronification in pancreatic cancer

Central sensitization should not be regarded only as a downstream result of peripheral nerve injury, but should also be considered an important mechanism that actively maintains chronic and treatment-resistant pain in pancreatic cancer 26, 48, 66, 69, 71, 72. Continuous nociceptive input from pancreatic duct obstruction, perineural invasion, perineural neuritis, tumor-related inflammation, and persistent nerve injury can induce activity-dependent plasticity in the spinal cord and brain 17, 21, 60, 69, 71, 72, 75. Under this condition, pain may gradually change from localized visceral or neuropathic pain to a more amplified pain state, which is characterized by hyperalgesia, allodynia, recurrent breakthrough pain, and incomplete response to increasing doses of opioids 16, 22, 23, 36, 48, 66, 69.

At the spinal cord level, sustained input from primary afferent nerves increases glutamatergic transmission and promotes NMDA receptor-mediated sensitization of dorsal horn neurons. This process can make dorsal horn neurons more excitable and responsive to repeated stimulation 23, 26, 48, 66, 69, 75. At the same time, inhibitory control mediated by GABAergic and glycinergic neurons may be weakened, so that normally weak or non-painful stimuli can produce stronger pain responses 23, 66, 69, 71. In addition, activated microglia and astrocytes release inflammatory cytokines and neurotrophic factors, such as BDNF, which further increase neuronal excitability, promote disinhibition, and maintain maladaptive synaptic plasticity 48, 66, 69, 70.

Central sensitization in pancreatic cancer is also related to abnormal descending pain modulation. Brainstem pathways, especially descending facilitation from the rostral ventromedial medulla, may enhance spinal nociceptive transmission, whereas endogenous descending inhibition may become insufficient 48, 69, 71, 75, 76. At the supraspinal level, long-term nociceptive stimulation may cause structural and functional changes in pain-related brain regions, including the insula, anterior cingulate cortex, and prefrontal cortex 69, 75, 76, 182. These central changes may partly explain why pancreatic cancer pain can become more severe than expected from local tumor burden alone, and why peripheral treatments such as celiac plexus block or neurolysis may fail to provide complete and long-lasting pain relief once chronic pain has developed 23, 26, 44, 48, 66, 89.

## 7. Distinct contributions of the nervous system in PC-associated pain

Pancreatic cancer pain is regulated by interactions among sympathetic, parasympathetic, and sensory neural pathways, which contribute differently to pain generation and maintenance. These pathways participate in tumor-nerve crosstalk, neuroinflammation, peripheral sensitization, and pain chronification.

The sympathetic nervous system mainly acts as a pain-amplifying pathway. Through adrenergic signaling, sympathetic activation promotes neuroinflammation, enhances nociceptor excitability, and facilitates tumor-nerve interactions 83, 140, 183, 184. Sustained sympathetic activity may therefore contribute to persistent pain during tumor progression 185-188.

In contrast, the parasympathetic nervous system exerts predominantly modulatory effects. Vagal signaling suppresses inflammatory responses through cholinergic anti-inflammatory pathways and may attenuate pain hypersensitivity under certain conditions 189-192. However, its role in pancreatic cancer remains complex and appears to depend on the local tumor microenvironment, as cholinergic signaling may also influence tumor growth and neural remodeling 140, 141, 189.

Sensory afferent pathways represent the most direct neural mediators for pancreatic cancer pain. Sensory neurons transmit nociceptive signals from the pancreas to the central nervous system and undergo sensitization in response to neurotrophic factors, chemokines, and inflammatory mediators 24, 52, 68, 193. These changes promote neuronal hyperexcitability, neurogenic inflammation, neuropathic pain, and pain chronification 24, 52, 68, 193.

**Figure 6:**
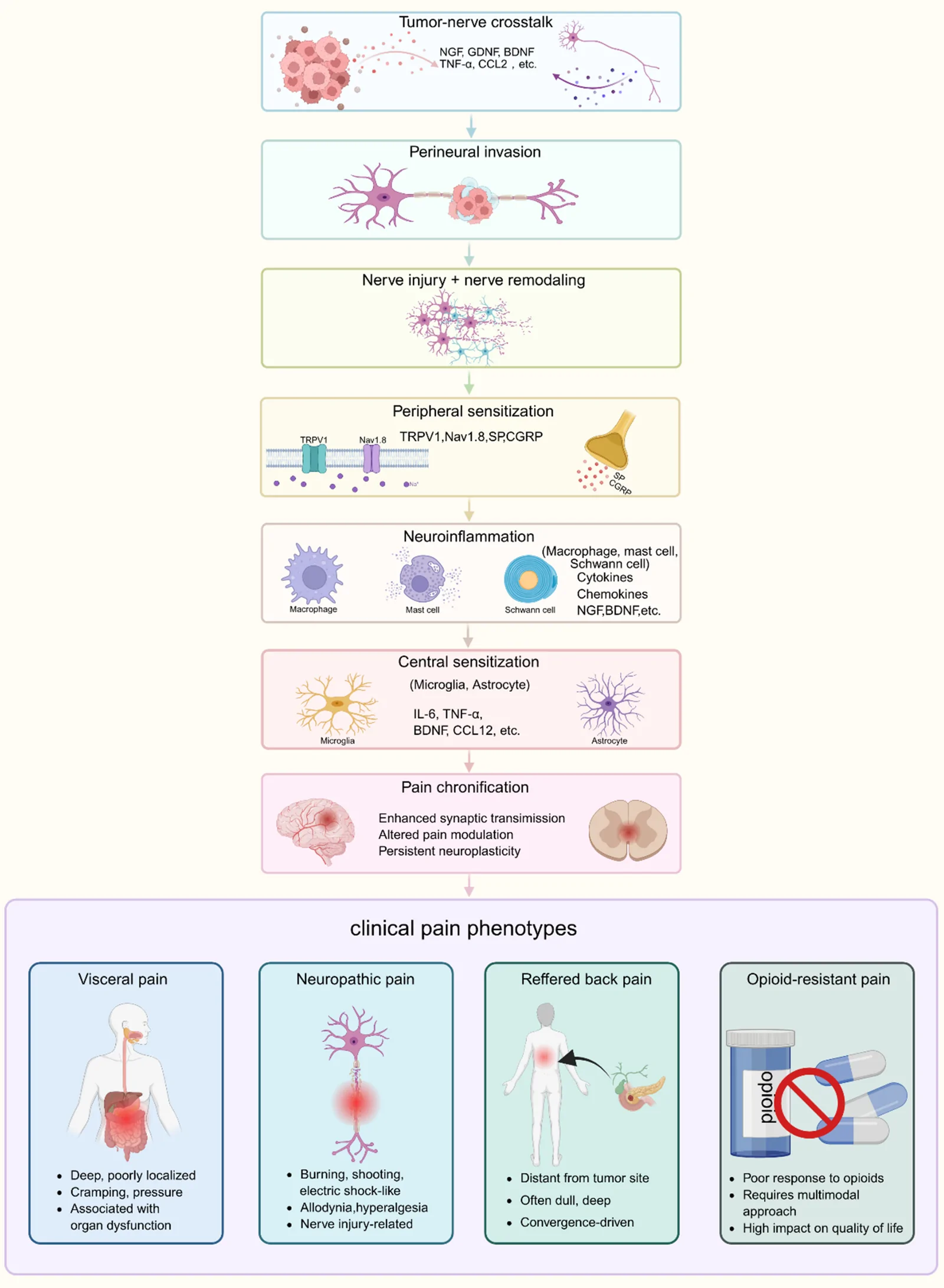
** A pain-centered conceptual framework for pancreatic cancer pain: from tumor‒nerve crosstalk to pain chronification.** Tumor-derived neurotrophic factors, inflammatory cytokines, and chemokines, such as NGF, GDNF, BDNF, TNF-α, CCL2, etc. initiate tumor-nerve crosstalk and promote perineural invasion. Subsequent nerve injury and nerve remodeling induce peripheral sensitization through the activation of nociceptive molecules, including TRPV1, Nav1.8, substance P, and CGRP. At the same time, macrophages, mast cells, Schwann cells, cytokines, chemokines, and neurotrophins contribute to sustained neuroinflammation. Persistent peripheral input and neuroimmune activation further induce central sensitization, which is associated with microglial and astrocytic activation and increased production of IL-6, TNF-α, BDNF, and related mediators. These changes finally lead to pain chronification, characterized by enhanced synaptic transmission, altered pain modulation, and persistent neuroplasticity. Clinically, this process may manifest as different pain phenotypes, including visceral pain, neuropathic pain, referred back pain, and opioid-resistant pain. These pain phenotypes are not entirely distinct, but often overlap and change dynamically as the disease progresses.

Overall, the sympathetic nervous system mainly promotes pain through adrenergic and inflammatory mechanisms, whereas the parasympathetic nervous system exerts regulatory effects that vary with the local microenvironment 139, 140, 185, 189. Sensory neurons serve as the primary pathway for nociceptive signal transmission 26, 48, 61, 87, 194. These neural pathways contribute to pain initiation, maintenance, and chronification in pancreatic cancer 24, 52, 83, 140, 189, 195 (Figure 6).

## 8. Conventional pain management in PC

Pain associated with PC is especially hard to treat due to the combined effects of neural invasion, inflammation and central sensitization 12. Current management is based on a multimodal and stepwise approach with pharmacological therapies followed by interventional and supportive measures. Despite the various options available, pain relief is often incomplete and adverse effects of treatment often limit adherence and overall tolerability 12, 35, 39.

### 8.1 Systemic pharmacological therapy

Systemic pharmacological therapy is one of the cornerstones of pain therapy in PC, as the majority of patients have a mixed nociceptive and neuropathic pain. A multimodal, stepwise and patient tailored approach is recommended, combining systemic analgesics with anticancer treatments, with interventional procedures, such as celiac plexus block or neurolysis, when indicated 16. Management is usually started with non-opioid analgesics in cases of mild pain and progresses to opioids in cases of moderate to severe symptoms 196. Opioid treatment is typically started with immediate-release preparations to determine an effective dose, and then switched to sustained-release preparations for baseline analgesia, with short-acting preparations reserved for breakthrough pain 196, 197.

#### 8.1.1 Non-opioid analgesics

##### Acetaminophen

Acetaminophen is generally considered a first-line agent in the treatment of mild cancer-related pain and as a baseline agent used in conjunction with opioids as pain intensity increases 198. Its centrally mediated analgesic effects include inhibition of prostaglandin synthesis and activation of descending inhibitory pathways and it has a more favourable gastrointestinal safety profile than NSAIDs 199. For long-term use, most guidelines recommend a maximum daily dose of 3 g, with an absolute maximum of 4 g/day, and dose reduction in patients with hepatic impairment, malnutrition or concomitant hepatotoxic medications 198. Although inadequate as a sole treatment for advanced PC, acetaminophen is a rational, low toxicity component of multimodal analgesic regimens.

##### NSAIDs

NSAIDs can be considered for mild to moderate pain or as adjuncts to opioids, especially in cases where inflammatory, capsular, or musculoskeletal factors contribute to symptoms. However, evidence specific to PC is limited and adverse effects often limit long-term use 200. Given that biliary obstruction, impaired renal perfusion, malnutrition, thrombocytopenia and concomitant anticoagulation are very common in these patients, NSAID therapy can increase the risk of gastrointestinal bleeding, renal dysfunction and cardiovascular events 198. When used, the lowest effective dose for the shortest feasible duration of use of NSAIDs should be administered, preferably in combination with gastroprotective agents and under close clinical monitoring.

##### Adjuvant analgesics

Adjuvant analgesics are important in the management of the neuropathic and mixed mechanism pain prevalent in PC because of the involvement of nerves and plexus. Neuropathic pain, which is often described as burning, shooting, or electric shock-like pain, and associated with celiac plexus or nerve root infiltration, commonly treated first-line with anticonvulsants like gabapentin or pregabalin. These agents require slow titration and renal dose adjustment with sedation and dizziness being the primary dose-limiting effects 198, 201. Antidepressants, such as tricyclic antidepressants (e.g. amitriptyline, nortriptyline) and serotonin-NE reuptake inhibitors (e.g. duloxetine, venlafaxine), both increase descending inhibitory pathways, improve neuropathic symptoms, and may also improve sleep, anxiety, and depression 198. Their use needs careful consideration of anticholinergic burden, cardiovascular risk, and drug-drug interactions, especially in frail or polymedicated patients 198.

Corticosteroids, most commonly dexamethasone, are occasionally used for PC-related pain in which peritumoral inflammation, edema, capsular stretch or nerve pressure is suspected. They may offer short-term analgesia and temporary improvements in appetite and well-being 202, but benefits are low and short-lived, and adverse effects, such as hyperglycemia, risk of infection, myopathy, osteoporosis, fluid retention, gastrointestinal toxicity, mood and sleep disturbances, and suppression of the hypothalamic-pituitary-adrenal axis, limit their use 202. Accordingly, corticosteroids should be limited to short courses of carefully monitored therapy in selected patients 202.

Additional adjuvant analgesics, such as topical or systemic local anaesthetics (e.g. lidocaine patches), N-methyl-D-aspartate (NMDA) receptor antagonists such as ketamine, α2-adrenergic agonists, and bone-targeted agents in the presence of skeletal metastases, are generally reserved for patients with refractory or complex pain syndromes, and should be initiated with specialist palliative or pain management supervision 198. Mechanism-based approaches, such as pancreatic enzyme replacement therapy in people with exocrine insufficiency, may also decrease postprandial discomfort, providing an indirect contribution to overall pain control 16.

#### 8.1.2 Opioids

Opioids are the mainstay pharmacologic treatment for moderate to severe PC-related pain, and are often necessary throughout the course of the disease for the treatment of both visceral and neuropathic components. In opioid naive patients, treatment is usually started with immediate release tramadol or low doses of strong opioids (eg, hydrocodone, morphine, oxycodone, oxymorphone, hydromorphone) as needed to establish individual requirements. Once a stable dose is achieved on a daily basis, patients are switched to extended-release formulations with rapid release opioids (around 10 to 20% of the total 24-hour dose) reserved for breakthrough pain 73, 74, 203. Most opioids are µ-receptor agonists, which block ascending nociceptive pathways and stimulate descending inhibitory mechanisms. Agents such as methadone, levorphanol, tramadol and tapentadol have the additional properties of antagonizing the N-methyl-D-aspartate receptor or inhibiting monoamine reuptake and may be beneficial in complex or neuropathic pain states 36, 74, 176. No one opioid is clearly superior, and selection should be individualized based on previous response, comorbidities, concomitant medications, and metabolic factors. Opioid rotation is the practice when analgesia is inadequate or toxicity occurs, typically by switching to an alternative opioid at 50-75% of equianalgesic dose to compensate for incomplete cross-tolerance 198. In patients with significant renal impairment, it is advisable to use fentanyl and methadone as they do not produce neurotoxic metabolites, and in hepatic dysfunction, a lower starting dose and longer dosing intervals are recommended 204-206.

On the other hand, because opioids are still indispensable in the management of advanced pancreatic cancer pain and are often used with increasing doses and duration, opioid-induced hyperalgesia (OIH) should be acknowledged as a possible factor contributing to worsening pain 16, 36, 48, 74, 176. OIH is different from pharmacological tolerance. Tolerance mainly refers to the gradual reduction of analgesic effect, whereas OIH means that patients become more sensitive to pain during opioid exposure 36, 74, 176, 204. In clinical practice, OIH may be characterized by generalized pain exacerbation, expansion of the painful area, pain triggered by normally non-painful stimuli, or paradoxical worsening of pain despite increasing opioid dose 36, 69, 74, 176. Mechanistically, OIH shares several features with central sensitization, including enhanced NMDA receptor activity, activation of spinal microglia and astrocytes, neuroinflammation, increased release of excitatory neuropeptides, and enhanced descending facilitation 36, 58, 66, 69, 71, 74, 176.

In pancreatic cancer, persistent peripheral nociceptive input from perineural invasion, inflammation, and nerve injury has already created a sensitized state 23, 24, 26, 48, 60, 63, 69. Under this condition, long-term or high-dose opioid exposure may further promote maladaptive plasticity and contribute to refractory pain and increasing opioid requirements 16, 22, 36, 74, 176. Therefore, worsening pain during opioid dose escalation should not be managed only by further increasing the opioid dose. Clinicians should carefully reassess the underlying mechanisms and causes of pain 17, 36, 176, 196, 197. Possible explanations include tumor progression, neuropathic transformation, breakthrough pain, opioid tolerance, and OIH-related pain amplification 15, 36, 73, 204. Recognition of OIH also supports the use of more individualized strategies, such as opioid rotation, combination with adjuvant analgesics for neuropathic or centralized pain, and selected NMDA receptor-modulating treatments under specialist supervision 16, 17, 36, 176, 196.

#### 8.1.3 Mechanisms responsible for the limited effect of conventional treatment on pancreatic cancer pain

Conventional treatments often provide incomplete pain relief in pancreatic cancer because they mainly block pain transmission or reduce symptoms, but cannot sufficiently inhibit the biological mechanisms that continuously generate pain 16, 17, 24-26, 44, 48. Opioids can inhibit nociceptive signaling, but they cannot stop perineural invasion, perineural neuritis, Schwann cell reprogramming, chemokine and neurotrophin signaling, or ongoing tumor progression 15, 23, 26, 48, 52, 60, 63, 65. Celiac plexus neurolysis can block important visceral afferent pathways, but it does not reverse distal neural invasion, central sensitization, or widespread neuroimmune activation 23, 26, 44, 48, 60, 88, 89, 91. Systemic anticancer therapy may reduce tumor burden and temporarily improve pain, but this effect may be weakened by chemotherapy-induced peripheral neuropathy, repeated neural injury, or disease progression 15-17, 23, 39, 48.

After pain chronification occurs, treatment resistance may be further reinforced by spinal and supraspinal plasticity, impaired descending inhibitory control, recurrent breakthrough pain, and opioid-induced hyperalgesia in some patients 16, 36, 48, 69, 71, 72, 176. These mechanisms may explain why many patients only obtain partial or short-term pain relief and why the requirement for multimodal analgesic treatment often increases during disease progression 15-17, 22, 26, 44, 196, 197. Therefore, the limited efficacy of conventional treatment should not be understood only as a problem of drug dose or analgesic selection, but also as a result of persistent tumor-nerve interaction, sustained neuroimmune activation, and central sensitization.

### 8.2 Disease-modifying therapy

Systemic chemotherapy, although most likely to be aimed at controlling tumor growth, can decrease tumor burden and PNI and thus provide partial pain relief 35, 39. This analgesic effect is often transient and may be offset by treatment-related neurotoxicity. Agents such as oxaliplatin and irinotecan may induce chemotherapy-induced peripheral neuropathy, which is characterized by burning, tingling or numbness that may be persistent and paradoxically increase the overall pain 35. Consequently, pain relief by tumor regression is often limited by the development of neuropathic complications.

### 8.3 Interventional therapies

#### 8.3.1 Celiac Plexus Neurolysis (CPN)

Endoscopic ultrasound-guided or percutaneous CPN is one of the most effective interventions in decreasing PC-related pain and opioid needs 88. CPN produces analgesic effects mainly by interrupting visceral afferent transmission 25, 29, 44, 87-89, 91. Through chemical or thermal ablation of the celiac plexus, this procedure can block sympathetic and nociceptive signals from the pancreas, thereby providing relatively rapid pain relief 25, 29, 44, 87-89, 91. However, its analgesic benefit is often transient, usually lasting only 2-3 months in many patients 89, 91, 207. Adverse events include orthostatic hypotension, diarrhea and bleeding, with rare but severe neurological complications such as paraplegia due to inadvertent spread of neurolytic agents 44, 90. In addition, the efficacy of CPN is dependent on tumor location and degree of celiac plexus invasion, with reduced pain relief in patients with extensive metastatic disease 208.

#### 8.3.2 Celiac Plexus Irradiation

Celiac plexus directed irradiation is one neurotargeted option for refractory upper abdominal pain in pancreatic and other upper gastrointestinal malignancies. Implantation of iodine-125 seeds around the celiac ganglion is able to provide prolonged analgesia but requires advanced imaging, precise dosimetry, and experienced operators; the potential complications include radiation-induced fibrosis and delayed neuropathic pain, which limit widespread use 43, 209, 210. More recently, single-fraction stereotactic celiac plexus radiosurgery has become an alternative noninvasive treatment. Phase II studies show significant pain relief with acceptable toxicity in selected patients, supporting its use as an adjunct to standard pharmacologic and interventional therapies, although additional comparative trials are needed 210.

#### 8.3.3 Thoracoscopic Splanchnicectomy (TS)

TS is a minimally invasive procedure in which the thoracic splanchnic nerves are interrupted and can result in significant, often durable pain relief with reduced opioid requirements in selected patients with unresectable PC 211, 212. The procedure involves general anesthesia, single-lung ventilation, and specialized thoracoscopic skills. Potential complications include pneumothorax, bleeding, infection, intercostal neuralgia, orthostatic symptoms, and chronic diarrhea due to sympathetic denervation 211, 213. Accordingly, TS should be reserved for carefully selected patients with refractory pain and adequate performance status, rather than patients with advanced frailty or significant cardiopulmonary compromise 214.

#### 8.3.4 High-Intensity Focused Ultrasound (HIFU)

HIFU is a noninvasive, image-guided therapeutic approach in which focused acoustic energy is delivered to pancreatic tumors and surrounding neural plexuses to thermally damage nociceptive pathways. Thermal coagulation of local neural structures may interrupt pain transmission within the tumor region 42, 43, 215-217. Clinical studies have reported rapid pain relief and decrease the need for opioids in a significant percentage of patients with advanced or unresectable PC with a generally low rate of severe complications 215-218. However, its effectiveness relies on sufficient access to the acoustic system and operator skills and may be constrained by bowel gas, stents, penetration depth, and the possibility of some or all analgesia being partial or transient 215, 216.

#### 8.3.5 Intrathecal Drug Delivery Systems (IDDS)

IDDS allow continuous delivery of concentrated analgesic agents into the cerebrospinal fluid, delivering effective segmental analgesia with significantly less systemic opioid exposure in carefully selected patients with refractory PC-related pain 219, 220. IDDS delivers opioids or other analgesic agents directly into the cerebrospinal fluid, allowing relatively high drug concentrations to be achieved in the central nervous system while reducing the required systemic dose 219-222. Observational studies and registry data, including PC-specific cohorts, show sustained pain relief, decreased systemic opioid requirements and stabilization or improvement in quality of life, with acceptable complication rates 219-222. However, IDDs require surgical implantation, specialized long-term follow-up and multidisciplinary expertise. Potential complications include infection, obstruction or migration of the catheter, granuloma formation, pump/programming failure, and the financial and logistical burden of maintenance. Consequently, IDDS should be used as an advanced and resource-intensive option for selected patients rather than routine use 219.

### 8.4 Palliative radiation therapy

Palliative radiotherapy gives relief from pain in about 60-100% of patients especially in case of locally advanced PC 42, 223, 224. Palliative radiotherapy can relieve pain mainly by reducing tumor burden and attenuating local inflammatory responses 42, 223, 224. Through irradiation of the tumor mass or nerve-invaded tissue, it may decrease nociceptive input from the pancreatic lesion 42, 223, 224. However, the analgesic effect may be temporary when tumor progression continues 42, 223, 224. Common acute adverse effects include fatigue, nausea, and enteritis 42, 223, 224. In addition, high-dose stereotactic body radiotherapy may cause severe gastrointestinal complications, including duodenal ulceration and gastrointestinal bleeding 42, 223, 224. Thus, although radiotherapy is effective in the local control of the tumor, it is less suitable for rapidly progressive pain.

### 8.5 Complementary therapies

#### 8.5.1 Acupuncture

Acupuncture and electroacupuncture, including transcutaneous electrical acupoint stimulation have been reported to relieve visceral and neuropathic pain in experimental and clinical studies 225-228. Their analgesic effects are believed to be mediated by the modulation of the autonomic nervous system and the stimulation of endogenous opioid release, especially enkephalins and dynorphins 225-228. These approaches are generally safe, low cost and may help to improve quality of life by reducing the opioid requirement 225-228. However, reported outcomes are heterogeneous because of variability in study quality, sample size and techniques. Larger, properly designed clinical trials are necessary to confirm efficacy and to establish protocols.

#### 8.5.2 Transcutaneous Electrical Nerve Stimulation (TENS)

**Figure 7:**
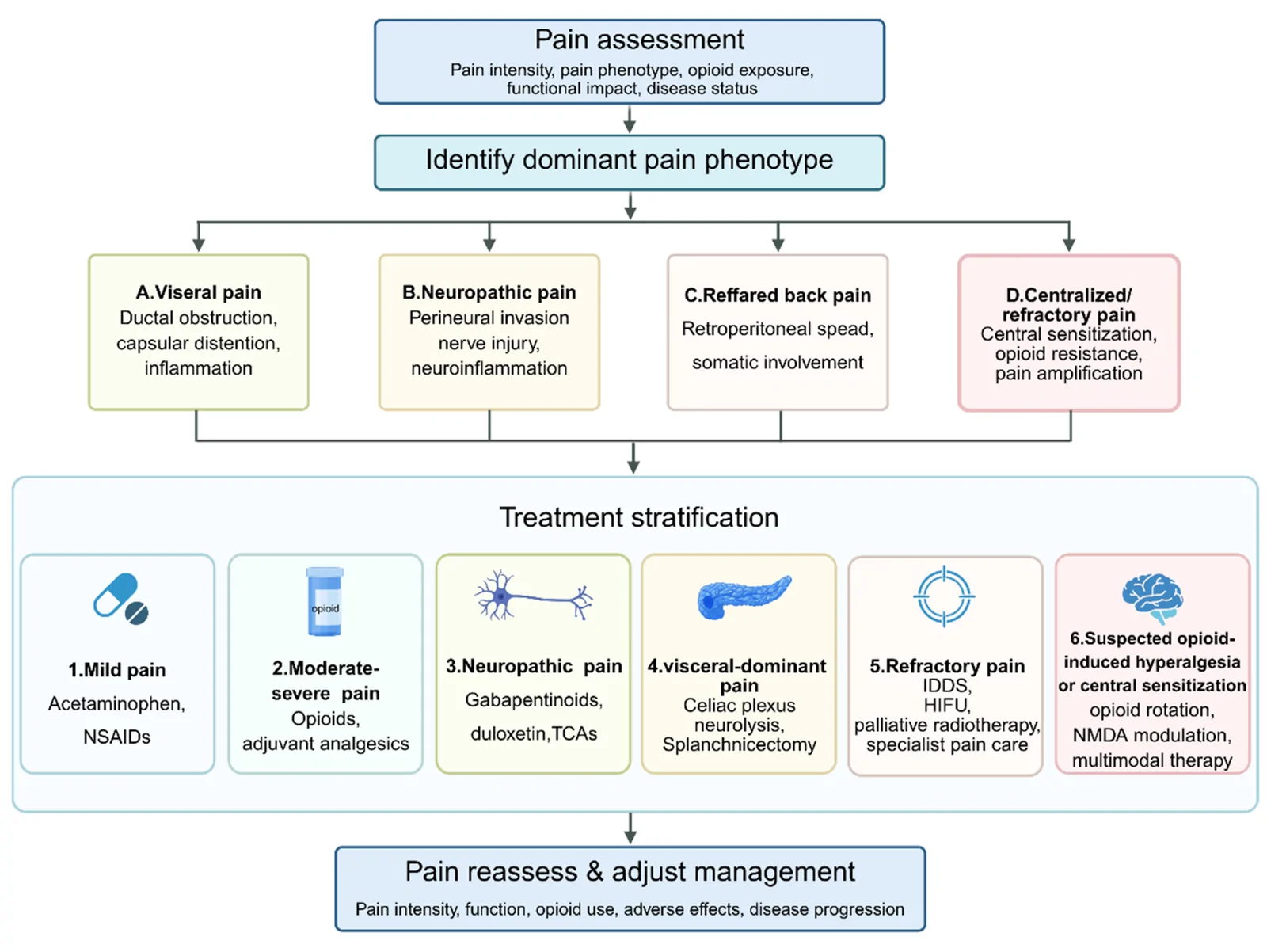
** Algorithmic management of pancreatic cancer pain.** This flowchart presents a phenotype-oriented approach to the assessment and management of pancreatic cancer pain. Pain assessment should include pain intensity, dominant pain phenotype, opioid use history, functional impact, and disease status. Based on the main clinical presentation, pancreatic cancer pain can be classified as visceral pain, neuropathic pain, referred back pain, or centralized/refractory pain. Treatment selection should be based on pain severity and phenotype. Mild pain may be managed with acetaminophen with or without NSAIDs. Moderate to severe pain usually requires opioids combined with adjuvant analgesics. Neuropathic features may indicate use of gabapentinoids, duloxetine, or tricyclic antidepressants (TCAs). Visceral-dominant pain may benefit from celiac plexus neurolysis (CPN), splanchnic block, or radiotherapy. Refractory pain may require intrathecal drug delivery systems (IDDS), high-intensity focused ultrasound (HIFU), radiosurgery, or specialist pain care. If opioid-induced hyperalgesia or central sensitization is suspected, opioid rotation, NMDA-receptor modulation, and multimodal therapy should be considered. Pain intensity, function, opioid use, adverse effects, and disease progression should be reassessed regularly to guide treatment adjustment. Abbreviations: NSAIDs, nonsteroidal anti-inflammatory drugs; TCAs, tricyclic antidepressants; IDDS, intrathecal drug delivery systems; HIFU, high-intensity focused ultrasound; NMDA, N-methyl-D-aspartate; CPN, celiac plexus neurolysis.

TENS is effective for short-term analgesia of PC pain and pain reductions of up to 78% immediately after treatment and up to 3 weeks without increased opioid consumption 229. It may also relieve associated symptoms such as constipation and appetite loss and is safe, noninvasive and generally well tolerated 229. Its analgesic effects are probably mediated by the modulation of spinal nociceptive transmission and activation of endogenous opioid pathways. However, the benefit is usually temporary, often only lasting hours, which means they need to be applied again and again 229 (Figure 7).

## 9. Emerging therapies for pancreatic cancer pain: evidence level, translational barriers, and limitations

Emerging therapeutic strategies for pancreatic cancer pain should be discussed according to the current level of evidence. For most of the approaches described below, the biological rationale is reasonable, but the supporting evidences are still mainly from preclinical studies, indirect observations, tumor-related studies, or studies that did not use pain relief as the primary endpoint. In many cases, the possible analgesic effect is inferred from reduced perineural invasion, decreased neural remodeling, or reduced tumor burden, rather than confirmed by well-designed clinical trials specifically focused on pancreatic cancer pain 16, 48, 49, 55, 68, 98, 174, 230. Therefore, these strategies should be regarded as investigational approaches at present, rather than established treatments. Several important translational challenges also need to be considered, including poor drug delivery caused by dense pancreatic stroma, tumor heterogeneity, lack of phenotype-based patient selection, potential off-target toxicity, and insufficient use of pain-related endpoints in oncology trials 16, 48, 231-233.

### 9.1 Therapeutic avenues for pain in PC induced by the nervous system

As mechanistic understanding of PC-related pain continues to progress, a new class of mechanism-based strategies has emerged that seeks to not only palliate symptoms, but also to interrupt the pathological interactions between cancer cells, neuronal structures and the stromal niche. Preclinical and early translational research has highlighted the role of PNI, neuroimmune activation, dysregulated adrenergic signaling, and maladaptive neural plasticity in the desmoplastic stroma as important contributors to persistent nociception and tumor progression. These findings offer a strong rationale for targeting specific molecular pathways, such as neurotrophic and chemokine signaling, neuroimmune crosstalk and β-adrenergic activity, and developing precision delivery platforms, such as neuron-targeted liposomal formulations, as effective adjuvants to conventional analgesic and antineoplastic therapies.

#### 9.1.1 Emerging therapies targeting PNI

Emerging therapeutic strategies against PNI in PC are increasingly focusing on the disruption of molecular crosstalk between tumor cells and nerves that drives tumor dissemination and neuropathic pain. A central approach involves blocking neurotrophic signaling, especially NGF-TrkA, NGF-TRPV1, GDNF-RET, and ARTN-GFRα1 that mediate neurite outgrowth, Schwann cell activation and sensory neuron sensitization 50, 86, 96, 98, 110, 169, 172, 173, 175. Inhibition of the NGF-TrkA axis by monoclonal antibodies (e.g. tanezumab) or small molecule TrkA inhibitors (e.g. PHA-848125) has been shown to decrease PNI-associated pain and neuronal hyperexcitability in preclinical settings 172, 173. Extending this concept, nerve-targeted nanotherapeutic platforms that deliver Trk inhibitors directly to intratumoral nerves have been shown to interfere with neurotrophin-Trk-mediated tumor-nerve interactions, reduce nerve density and PNI, and modulate the immune microenvironment, providing proof-of-principle for precision neural blockade 174. In addition, highly selective pan-Trk inhibitors, such as larotrectinib, which produce durable tumor responses and pain relief in NTRK fusion-positive solid tumors, provide evidence of the clinical feasibility of prolonged Trk inhibition 234, 235. As direct clinical evidence in PC is still lacking, preclinical evidence suggests that TrkA blockade may be a rational approach to restrain PNI and alleviate neuropathic pain.

However, most evidence for TrkA-centered analgesic strategies in pancreatic cancer remains preclinical or indirect. Their clinical use for pancreatic cancer pain will require trials that include pain intensity, opioid consumption, neuropathic pain scores, and quality of life as predefined endpoints.

#### 9.1.2 Potential therapy targeting neuroinflammation

Neuroinflammation is a major mechanism underlying persistent pain sensitization in PC, which is mediated by immune cell infiltration and proinflammatory mediators released in the perineural microenvironment 60, 132. Chemokine pathways such as CX3CL1-CX3CR1 and CXCL12-CXCR4 promote the recruitment of macrophages and microglia and directly contribute to neuronal hyper-excitability; pharmacologic inhibition of these receptors reduces perineural inflammation and mechanical allodynia in experimental models 236-238. Within this inflammatory environment, TNF-α is a key amplifier of pain signaling. Predominantly produced by tumor-associated macrophages and other myeloid cells, TNF-α perpetuates chronic inflammatory signaling, induces neural remodeling, and increases nociceptor sensitization 239, 240. Emerging TNF-α targeted approaches, such as depletion or functional inhibition of TNF-α producing macrophages and modulation of downstream signaling pathways, have been demonstrated to be efficacious in preclinical PC models by reducing perineural inflammation, immune-stromal niche remodeling, and tumor-associated pain 239-241. Alongside interventions targeting IL-6 and other proinflammatory cytokines, these results identify neuroinflammatory pathways as mechanistically plausible therapeutic targets to interrupt the self-perpetuating cycle of pain amplification and achieve more durable analgesia in PC 60, 239-241.

#### 9.1.3 Emerging therapy targeting sympathetic nerve activation

Sympathetic hyperactivation plays a major role in PC pain, mainly by neural excitation mediated by the β₂-adrenergic receptor 186, 188, 242. Adrenergic signaling enhances release of NE, nociceptor sensitization, and boosts neuroinflammation in the tumor microenvironment. Pharmacologic β-blockade, such as propanolol, decreases sympathetic tone and enhances the analgesic effect in both preclinical and clinical studies 186, 188. Targeted liposomal delivery of adrenergic antagonists further allows selective modulation of the tumor-nerve interface with minimal systemic toxicity and effective pain reduction 184. Chronic stress-induced adrenergic activation worsens pain by stimulating PNI and glial reactivity, suggesting that simultaneous inhibition of the β₂-adrenergic and neuroinflammatory pathways may have synergistic analgesic activity 188. Together, these findings identify sympathetic modulation as a potential and underappreciated approach to relieving neuropathic and stress-aggravated PC pain.

Preclinical studies have also shown that duloxetine has both analgesic and antitumor effects in PC 230. In KPPC mouse models, oral duloxetine had a significant analgesic effect; this effect was reversed by atipamezole, an α₂-adrenergic antagonist, suggesting that analgesia occurs primarily through enhancement of descending noradrenergic rather than serotonergic pathways 230. Concurrently, duloxetine suppressed proliferation, migration, and invasion of PC cells and CAFs, decreased Ki-67 indices, and rewrote the tumor microenvironment in an antitumor inflammatory direction, with reduced tumor-associated neutrophils and M2-like TAMs and increased M1-like TAMs. Duloxetine also modulated cytokines associated with cachexia (↓TNF-α, ↓IGFBP-3, ↑IGFBP-5/6), decreased muscle and adipose wasting, and extended survival in tumor bearing mice 230. These multimodal effects make duloxetine an emerging candidate for the simultaneous management of PC-associated pain and tumor progression.

#### 9.1.4 Novel treatment targeting Sensory nerves

Sensory neurons are now being recognized as active contributors to PC pain and tumor-neural crosstalk, with hematopoietic CSFs becoming important sensory-directed targets. G-CSF and GM-CSF are overexpressed in pancreatic adenocarcinoma and have non-hematopoietic action by activating receptor on hypertrophic pancreatic nerves and primary sensory neurons and cause peripheral sensitization, neurite sprouting, and amplification of nociceptive signaling 195. In preclinical models, neutralization of G-CSFR or GM-CSFRα, inhibition of downstream JAK-STAT or ERK signaling, or selective knockdown of GM-CSFRα in sensory neurons significantly decreases tumor-induced hyperalgesia and pathological nerve remodeling, in some cases without affecting tumor burden. These findings identify CSF signaling in sensory neurons as a preclinically promising mechanism-based target for localized or neuron-directed analgesia with potentially reduced systemic toxicity 195.

Targeting the pathways involved in neurotransmitters is another sensory-focused approach to PC pain. Inhibition of SP/NK1R and CGRP signaling interferes with neuropeptide-mediated tumor-nerve crosstalk and neurogenic sensitization, which reduce PNI-associated neuropathic pain 58, 243. NK1R antagonists, such as aprepitant, inhibit SP-mediated invasion of PC cells and perineural signaling, and have antitumor and analgesic properties, providing support for their evaluation in refractory PC pain 143. Similarly, CGRP receptor antagonists and anti-CGRP antibodies, effective in other chronic pain disorders, may be effective in reducing PC pain by limiting CGRP-dependent peripheral sensitization and neurogenic inflammation, although direct evidence in PC is limited 144. Collectively, these approaches provide a mechanism-based approach for precision analgesia by direct targeting of sensory neurons with the potential to minimize systemic adverse effects.

#### 9.1.5 Neuron-inhibitory liposomes for analgesia-focused neuromodulation

A liposomal delivery system targeting collagen and with a pH-responsive release has been developed to deliver lidocaine directly into the desmoplastic stroma of pancreatic tumors, in order to decrease nerve-tumor interactions that are responsible for neuropathic pain 232. These liposomes contain a collagen-binding peptide, which allows preferential accumulation inside the collagen-rich ECM and controlled lidocaine release in acidic conditions 231, 233, 244. Preclinical in vitro and orthotopic models have shown neural selectivity, less intratumoral innervation, inhibition of tumor growth, and less mesenteric metastasis, with no systemic toxicity 232. This neuron-inhibitory liposomal strategy is a targeted neuromodulatory strategy with the potential to simultaneously relieve pain and reduce disease progression.

Most emerging analgesic strategies for pancreatic cancer remain supported mainly by preclinical evidence. Duloxetine, cannabinoids, and targeted neuroinhibitory liposomes have shown potential analgesic effects in experimental models, but their clinical efficacy has not been confirmed 16, 25, 48. Further well-designed clinical trials are needed to clarify optimal dosing, nerve-targeted delivery, and safety before clinical application.

### 9.2 Cannabinoid-based therapy for PC pain

Cannabinoids are an emerging adjunct in the management of PC pain through modulation of both nociceptive and inflammatory pathways. Activation of CB1 receptors results in the inhibition of central nociceptive transmission, while activation of CB2 receptors reduces immune-mediated neuroinflammation 245, 246. Preclinical research indicates that cannabinoids, such as THC, CBD and HU-210, are effective in reducing pain by reducing neuronal hyperexcitability and release of inflammatory cytokines 247. Mechanistically, CB1 stimulation inhibits glutamate and SP release while CB2 stimulation inhibits macrophage and mast cell activity, which collectively inhibits pain signaling 245-247. Clinical observations of benefits include refractory pain and decreased opioid requirements 248. However, there are challenges, such as psychoactive effects, variability in formulation and lack of standardised dosing 249. Selective CB2 agonists have analgesic efficacy without central adverse effects and may be a safer choice for long-term administration 249. Overall, cannabinoid-based therapies offer a multimodal approach that includes the combination of direct nociceptive inhibition and neuroimmune modulation in the management of PC pain 249.

### 9.3 TGF-β pathway inhibition of pain relief in PC

Aberrant TGF-β signaling in PC is involved in desmoplasia, neural remodeling and pain sensitization 250, 251. Excessive TGF-β activity is responsible for the development of fibrosis and nerve entrapment, which worsens mechanical compression and chronic pain 250, 252, 253. It also induces PSCs to secrete NGF and other neurotrophins, creating a direct connection between stromal fibrosis and neuropathic pain 251. Inhibition of TGF-β signaling decreases ECM deposition, relieves neural compression and inhibits neurogenic inflammation 254, 255. Experimental studies show that TGF-β1 blockage restores normal sensory neuron activity and reduces pain behaviors 254. Pharmacologic inhibitors such as galunisertib and vactosertib (TGF-β receptor I kinase inhibitors) have been shown to be early-stage in reducing neural inflammation and improving pain outcomes 255. By reducing the fibrosis-induced compression of the nerves, TGF-β is an indirect but clinically relevant target for pain relief in advanced PC 251, 255.

### 9.4 mRNA-based therapeutics for pain in PC

Pain in PC is mainly caused by tumor invasion and nerve compression in a pro-inflammatory microenvironment [93]. Although mRNA vaccines are mainly developed for tumor control, they may also play a role in pain relief by decreasing tumor burden and PNI through the enhanced immune-mediated cytotoxicity [92, 93]. By modulating the tumor microenvironment, such as suppression of inflammation and fibrosis, the vaccine-induced immune responses may reduce cytokine-driven nociceptor sensitization and nerve irritation [108]. In addition, inhibition of tumor cells that are actively involved in nerve signaling may indirectly interfere with pain-promoting tumor-nerve crosstalk [92]. Although still in an early phase of studies, mRNA-based immunotherapy is a potential strategy to relieve PC-related pain besides its antitumor effects [92, 93, 108]. At present, the analgesic role of mRNA-based therapy in pancreatic cancer should be regarded as indirect and hypothetical. Future studies need to determine whether tumor-directed immune responses can actually reduce pain intensity, neuropathic symptoms, opioid requirements, or central sensitization-related outcomes (Table 1).

### 9.5 Candidate biomarkers and translational opportunities

A major translational gap in pancreatic cancer pain is the absence of validated biomarkers that reflect pain mechanisms rather than tumor burden alone 16, 25, 26, 48. Candidate biomarkers worth discussing include neurotrophic mediators such as NGF, GDNF, and artemin; chemokine pathways including CXCL12/CXCR4 and CXCL10/CCL21; and markers of neural injury such as circulating neurofilament light chain 32, 48, 49, 52, 53, 63, 109. Imaging biomarkers may also prove informative, particularly radiologic evidence of celiac plexus involvement, retroperitoneal neural invasion, nerve hypertrophy, or MRI-based features suggestive of perineural spread 8, 11, 33, 48, 80, 87. However, most of these candidates remain exploratory and have not yet been prospectively validated for pain phenotyping, treatment selection, or response monitoring in pancreatic cancer 48, 52. Accordingly, a biomarker section would be best framed as an emerging research priority rather than a clinically established tool.

At present, these strategies should still be considered hypothesis-generating, because pancreatic cancer-specific clinical trials focusing on analgesic outcomes are still lacking. In addition, their efficacy in pancreatic cancer pain cannot be directly inferred from studies in other chronic pain conditions.

## 10. Conclusions and Prospects

In conclusion, accumulating evidences suggest that pancreatic cancer pain is not merely a consequence of tumor burden but a multifactorial neurobiological process involving nociceptive, neuropathic, and chronic pain mechanisms. Ductal obstruction, inflammation, and tissue distortion contribute to pain initiation, whereas tumor-nerve interactions, perineural invasion, and neuroinflammation drive peripheral sensitization and neuropathic transformation. Persistent afferent input may subsequently induce central sensitization, promoting pain chronification. This pain-centered model provides an integrated framework for understanding pancreatic cancer pain and may facilitate the development of mechanism-based, phenotype-guided analgesic strategies.

Importantly, not all molecular pathways contribute equally to pancreatic cancer pain. Neurotrophic signaling pathways, chemokine networks, TRPV1 signaling, neuropeptides, Schwann cell activation, and neuroimmune interactions are likely to be directly involved in pain generation and maintenance through their effects on nociceptor sensitization, neural remodeling, and neuroinflammation 23, 24, 26, 49, 51-54, 56-60, 65-67, 79, 93, 102, 109. By contrast, mechanisms such as MUC1-MAG interactions, NAT10-mediated ac4C modification, and circRNA-associated neural adhesion pathways may act primarily as upstream facilitators of tumor-nerve interactions and neural invasion, while their direct contributions to specific pain phenotypes remain to be clarified 26, 48, 63, 65, 102, 105, 145, 146, 164.

Current treatment for pancreatic cancer pain is still mainly palliative and often does not sufficiently target the biological mechanisms that maintain pain. Future therapeutic strategies should therefore focus on mechanism-based interventions targeting key drivers of pain progression, including neurotrophic signaling, ion-channel activity, neuroimmune interactions, Schwann cell responses, and stromal remodeling. A pain-centered framework may help bridge molecular mechanisms and clinical pain phenotypes, thereby facilitating the development of more precise and effective analgesic strategies for pancreatic cancer pain.

## Figures and Tables

**Table 1:** Major molecular and neurobiological pathways relevant to pancreatic cancer pain: pain mechanisms, current evidence level, therapeutic implications, and limitations

Molecular pathway	Role in pain generation	Current evidence level	Potential therapeutic agents/strategies	Current limitations
NGF-TrkA/ p75^NTR^	Promotes neurite sprouting, nociceptor sensitization, TRPV1 upregulation, and downstream SP/CGRP release; contributes to hyperalgesia, abdominal pain, and neuropathic transformation	Strong preclinical; limited indirect/early clinical evidence. Pain relevance is strongly supported by mechanistic and animal studies, but pancreatic cancer pain-specific clinical trials are lacking	Anti-NGF antibodies; experimental TrkA inhibitors	Limited pancreatic cancer-specific clinical data; side effects and modest efficacy have restricted translation; pain endpoints are often not primary outcomes
GDNF-family ligands/RET (GDNF-GFRα1-RET; ARTN-GFRα3-RET)	Enhances sensory sprouting, neural hyperexcitability, and tumor-nerve invasion; mainly associated with neuropathic pain development and maintenance	Predominantly preclinical	Experimental RET-pathway inhibition or ligand-neutralizing strategies	No validated analgesic trials in pancreatic cancer; current data are mainly mechanistic or tumor-biology oriented rather than pain-directed
CXCL12/ CXCR4	Reinforces neurotrophin signaling, promotes neurite-tumor attraction, neuroinflammation, and mixed nociceptive-neuropathic pain	Preclinical plus limited translational/ clinical oncology evidence; pain-specific clinical evidence remains weak	CXCR4 antagonists (e.g., plerixafor-like strategies)	Most clinical studies focus on tumor control or immune modulation rather than analgesia; stromal barrier, tumor heterogeneity, and lack of pain phenotyping remain major obstacles
Sensory chemokine axes (CX3CL1/ CX3CR1; CXCL10/ CCL21-related signaling)	Promote directional migration toward nerves, sensory neuron recruitment, nerve hypertrophy, abdominal hypersensitivity, and radiating pain	Strong preclinical; limited correlative clinical evidence	Experimental chemokine receptor blockade	High pathway redundancy and compensatory signaling; no established analgesic therapy in pancreatic cancer
TRPV1	Lowers nociceptor activation threshold, increases Na<sup>+</sup>/Ca<sup>2+</sup> influx, amplifies neurogenic inflammation; linked to burning, treatment-resistant, and neuropathic pain	Strong preclinical; limited early clinical evidence	TRPV1 antagonists; desensitization-based strategies targeting TRPV1-positive nociceptors	Clinical translation has been limited by hyperthermia, thermosensory adverse effects, and modest efficacy; pancreatic cancer-specific trials are lacking
SP-NK1R	Amplifies neurogenic inflammation and tumor-nerve signaling; may contribute to persistent abdominal pain and neuropathic pain	Strong mechanistic/preclinical; limited indirect clinical evidence	NK1R antagonists (e.g., aprepitant-like strategies)	Current clinical experience is mainly from antiemetic use; direct analgesic benefit in pancreatic cancer remains unproven
CGRP-CGRP receptor	Enhances neuronal hyperexcitability, inflammatory amplification, and persistent nociceptor sensitization	Moderate-to-strong preclinical; indirect clinical relevance	Anti-CGRP antibodies; CGRP receptor antagonists	Current clinical evidence is largely extrapolated from other pain disorders such as migraine; role in pancreatic cancer pain remains insufficiently validated
TGF-β / PSC-CAF-stromal remodeling axis	Promotes fibrosis, neural compression, ECM remodeling, NGF reinforcement, and pain persistence; may contribute to deep chronic abdominal/ back pain	Preclinical plus indirect early clinical/oncology evidence	TGF-β pathway inhibitors (e.g., galunisertib-like strategies); stromal-targeting approaches	Analgesic effects are likely indirect; pleiotropic biology, toxicity concerns, and lack of pain-focused trials limit translation
Neuroimmune loop (mast cells, TAMs, inflammatory mediators)	Drives perineural neuritis, inflammatory amplification, spontaneous pain, hypersensitivity, and reduced analgesic responsiveness	Preclinical and clinicopathologic association; no dedicated analgesic trials	Mast cell stabilization; macrophage/chemokine-targeting strategies; broader neuroimmune modulation	Immune pathways are biologically broad and may cause off-target effects; no validated biomarkers or clinical trials specifically targeting pancreatic cancer pain
Central sensitization-related pathways (NMDA signaling, glial activation)	Maintain pain chronification, allodynia, breakthrough pain, refractory pain, and possible overlap with opioid-induced hyperalgesia	Strong biological rationale and clinical relevance; interventional evidence remains indirect	NMDA-modulating strategies (e.g., ketamine- or methadone-based approaches); opioid-sparing multimodal therapy; experimental glial modulators	Difficult patient selection; lack of robust biomarkers; potential CNS adverse effects; not specific to pancreatic cancer biology
